# Mixed-effects two-stage residual inclusion methods for individual patient data meta-analysis: A methodological framework for causal inference in survival analysis

**DOI:** 10.1017/rsm.2026.10103

**Published:** 2026-06-29

**Authors:** Heather Hufstedler, Alexander M. Danzer, Valentijn Marnix Theodoor de Jong, Till Bärnighausen

**Affiliations:** 1 Heidelberg Institute for Global Health, UniversitätsKlinikum Heidelberg, Germany; 2 Volkswirtschaftslehre, Katholische Universität Eichstätt-Ingolstadt, Germany; 3 Julius Center for Health Sciences and Primary Care, Utrecht University, Netherlands

**Keywords:** causal inference, individual patient data meta-analysis, instrumental variables, survival analysis, two-stage residual inclusion

## Abstract

Individual patient data meta-analyses (IPDMAs) provide powerful tools for synthesizing evidence across studies, yet methods for addressing unmeasured confounding in observational IPDMAs with survival outcomes are rarely implemented. Instrumental variable (IV) approaches offer causal inference capabilities but face practical challenges in hierarchical data structures, particularly the lack of standard diagnostics for instrument strength in nonlinear mixed-effects models. We adapt and evaluate a frequentist mixed-effects two-stage residual inclusion (2SRI) framework for survival IPDMAs, extending traditional IV methods to accommodate study-level and temporal clustering while handling time-to-event outcomes through Cox proportional hazards models. Because classical *F*-statistics are unavailable for logistic mixed-effects first-stage models, we propose the Wald $\chi^{2}$ statistic as a practical instrument-strength diagnostic and empirically characterize its relationship to estimator performance. Through a comprehensive simulation study with 48 scenarios—varying unmeasured confounding (weak to very strong), instrument–treatment association strength (0.3–1.0), and cross-study IV allocation patterns—we evaluated 2SRI against naive mixed-effects Cox models using bias, coverage, variance, and mean squared error. The design was anchored to realistic IPDMA structure (10 studies, N≈4,357 ) from pooled Ebola data, with 1,000 replications per scenario. Results show that under weak confounding, naive models dominate on all metrics. With moderate-to-strong confounding and realized Wald $\chi^{2}$ exceeding 150–200, mixed-effects 2SRI substantially reduces bias and achieves near-nominal coverage, though with inflated variance. We provide empirical guideposts linking realized first-stage strength to expected performance, enabling analysts to judge when 2SRI will outperform conventional approaches in hierarchical survival IPDMAs. All simulations assume a common treatment effect across studies. Performance under heterogeneous effects remains to be established.

## Highlights

### What is known?

- Quasi-experimental methods, including instrumental variable (IV) approaches, are underutilized in IPD meta analyses of observational data despite their increased utilization in single studies.
- One barrier to their implementation in pooled analyses is that the classical F-statistics are unavailable in mixed effects models, leaving analysts without a practical way to assess instrument strength in these settings.

### What is new?

- We adapt and evaluate a frequentist mixedeffects two-stage residual inclusion (2SRI) framework for survival IPD meta analyses, extending IV methods to handle study-level and temporal clustering with Cox proportional hazards models for time-to-event outcomes.
- We propose the Wald X2 statistic as a practical instrument-strength diagnostic and show that under weak confounding naive models dominate, while under moderate-to-strong confounding with realized Wald X2 exceeding 150–200, mixed-effects 2SRI substantially reduces bias and achieves nearnominal coverage.
- We provide empirical guideposts linking realized first-stage instrument strength to expected estimator performance, giving analysts decision rules for when mixed-effects 2SRI will outperform conventional approaches.

### Potential impact for RSM readers

- These guideposts offer a concrete diagnostic that may lower the practical barrier to adopting IV-based 2SRI methods in IPD meta analyses of observational data.

## 1 Introduction

In population and global health research, where the aim is to draw population-level conclusions, combining data from multiple studies to conduct a meta-analysis (MA) is an efficient and cost-effective approach compared to running new, large studies.^1^ This method of combining, or pooling, data increases statistical power and allows for the assessment and quantification of between-study heterogeneity in effects across various sites, settings, and populations. MAs are generally conducted using one of two approaches (or a combination of the two): pooling summary estimates from published studies, a traditional method referred to as an aggregate data MA (ADMA), or by obtaining individual-level patient data (IPD) from separate studies to perform an individual patient data MA (IPDMA). IPDMAs can be conducted using either a one-step approach, which combines the IPD into one dataset and analyzes it directly, or the two-step approach, which analyzes each study separately before combining the study-specific estimates in a traditional MA model, like an ADMA.^2^^,^ ^3^ IPDMAs can provide estimates similar to ADMAs, but IPDMAs have several benefits, including the ability to evaluate data quality (e.g., patterns of missingness and differences in how variables were measured),^4^ and to standardize analytic approaches (e.g., inclusion/exclusion criteria, handling of missing data, and covariate adjustment in standardized statistical models), thereby minimizing the impact of between-study heterogeneity and allowing for the investigation of effect modification at the individual level.^3^^,^ ^5^^,^ ^6^

Across disciplines, where randomization is often not feasible, quasi-experimental methods, such as difference-in-differences (DiD),^7^ interrupted time series (ITS),^8^ regression discontinuity designs (RDDs),^9^ and instrumental variables (IVs),^10^ have long been implemented to strengthen causal inference by exploiting naturally occurring or otherwise exogenous variation in treatment assignment or exposure, thereby addressing threats from measured and unmeasured confounding. These methods have been recently and increasingly utilized in medicine in single observational studies and RCTs,^11^^–^ ^17^ but have been seldom been applied to IPDMAs in health research.^18^^,^ ^19^ Barriers to implementing these methods in IPDMAs may arise from practical data issues from pooling data from studies that differ in sample sizes, recruitment periods, and site practices, and therefore presents additional challenges in satisfying the assumptions required by the methods across datasets. Other barriers include the lack of well-developed statistical methods, diagnostic procedures, and software tools for conducting such analyses in IPDMAs.

To address this gap, we adapt and evaluate a frequentist mixed-effects two-stage residual inclusion (2SRI) framework.^20^ We also examine how first-stage Wald statistics can serve as a practical diagnostic for instrument strength in nonlinear mixed-effects models, where the familiar linear-model F-test has no direct analog.

## 2 Mixed effects two-stage residual inclusion for IPDMA

### 2.1 Instrumental variable models

IV methods address unmeasured confounding by exploiting quasi-random variation in treatment assignment or exposure. A valid IV must satisfy three assumptions: (i) relevance (the IV predicts treatment), (ii) independence (the IV shares no unmeasured common causes with the outcome), and (iii) exclusion restriction (the IV affects the outcome only through treatment).^21^

**Figure 1:**
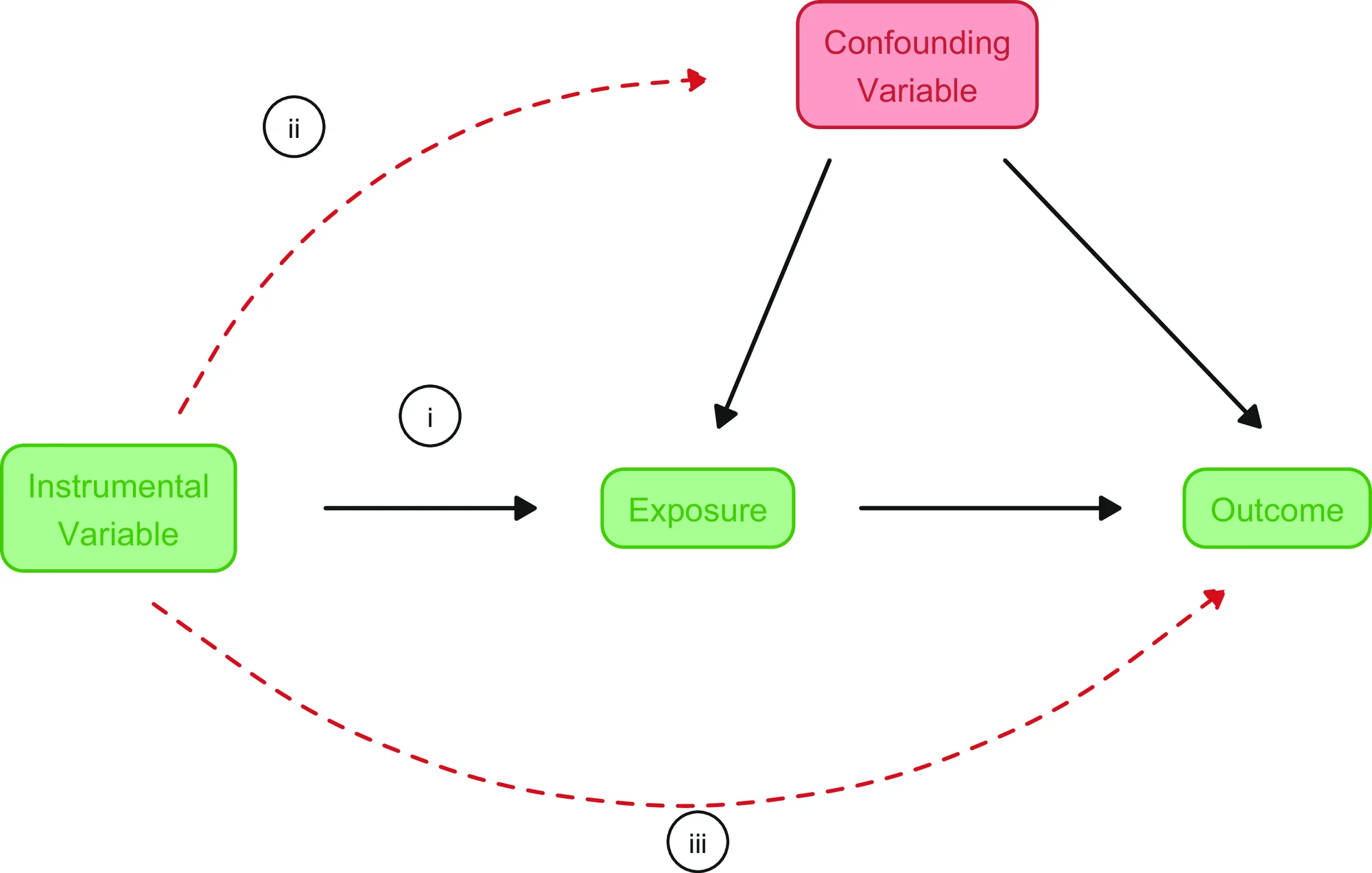
Directed acyclic graph (DAG) illustrating the three assumptions required for a valid instrumental variable (IV): (i) relevance, (ii) independence, and (iii) exclusion restriction. Dashed red arrows indicate the two pathways that must be assumed absent but cannot be empirically verified from observed data.

Figure 1 illustrates these assumptions as a directed acyclic graph (DAG). Of the three, only the relevance assumption (i) is directly testable: the strength of the instrument–exposure association can be evaluated using first-stage diagnostics, most commonly the partial *F*-statistic in linear models.^22^ The dashed red arrows depict the two pathways that must be assumed absent—a direct path from the instrument to unmeasured confounders (assumption ii, independence) and a direct path from the instrument to the outcome, bypassing treatment (assumption iii, exclusion restriction). Neither assumption (ii) nor assumption (iii) can be empirically verified from the observed data. The plausibility of both must therefore be defended through contextual reasoning and domain knowledge, including careful consideration of the processes through which the instrument is thought to operate, and careful consideration of alternative pathways through which the instrument could plausibly influence the outcome or correlate with unmeasured characteristics of individuals or study sites.

### 2.2 Mixed effects two-stage residual inclusion

In medicine, when the outcome of interest is survival, researchers conducting IPDMAs may adopt a mixed-effects Cox proportional hazards model in a one-step IPDMA.^23^

Formally, the naive model (without accounting for endogeneity) is

(2.1) $$\begin{array}{r} \log h_{ijk}(t)=\log h_{0}(t)+\beta_{T}T_{ijk}+\beta_{\text{X}}^{T}X_{ijk}+u_{j}^{\left(S\right)}+v_{k}^{\left(S\right)}, \end{array}$$

where $h_{0}(t)$ is the unspecified baseline hazard, $T_{ijk}$ is the treatment for individual *i* in study *j* entering at time *k* (e.g., month), $X_{ijk}$ is a vector of observed covariates, $\beta_{T}$ is the treatment effect, $\beta_{\text{X}}$ is a vector of covariate effects, and $\left(u_{j}^{\left(S\right)},v_{k}^{\left(S\right)}\right)$ are random intercepts for study and time. The random intercepts for study and time capture cluster-level heterogeneity in baseline risk; their interpretation in the context of survival IPDMAs is detailed in Section 3.2.

Among IV approaches, 2SRI is appropriate when the second-stage model is nonlinear, as in the Cox proportional hazards model for survival data, where alternative approaches, such as two-stage least squares (2SLS) or two-stage predictor substitution (2SPS), can be biased.^20^ In 2SRI, the first-stage models treatment as a function of the instrument and observed covariates, then residuals are calculated, and both treatment and residuals enter the second-stage survival model. Formally, the mixed-effects 2SRI model is estimated in two stages.

First, a mixed-effects logistic regression models treatment assignment:

(2.2) $$\begin{array}{r} \mathrm{logit}\mathrm{Pr}(T_{ijk}=1)=\alpha_{0}+\alpha_{1}Z_{ijk}+\alpha_{2}^{T}X_{ijk}+u_{j}^{\left(T\right)}+v_{k}^{\left(T\right)}, \end{array}$$

where $T_{ijk}$ is the treatment, $Z_{ijk}$ is the instrument, $X_{ijk}$ is the vector of observed covariates, and $\left(u_{j}^{\left(T\right)},v_{k}^{\left(T\right)}\right)$ are random effects for study and time.

The predicted probability is then estimated:

(2.3) $$\begin{array}{r} {\hat{p}}_{ijk}={\mathrm{logit}}^{-1}\;({\hat{\alpha }}_{0}+{\hat{\alpha }}_{1}Z_{ijk}+{\hat{\alpha }}_{2}^{T}X_{ijk}+{\hat{u}}_{j}^{\left(T\right)}+{\hat{v}}_{k}^{\left(T\right)}). \end{array}$$

The residuals are calculated:

(2.4) $$\begin{array}{r} {\hat{r}}_{ijk}=T_{ijk}-{\hat{p}}_{ijk}. \end{array}$$

The second stage is a mixed-effects Cox model with residuals:

(2.5) $$\begin{array}{r} \log h_{ijk}(t)=\log h_{0}(t)+\beta_{T}T_{ijk}+\delta {\hat{r}}_{ijk}+\beta_{\text{X}}^{T}X_{ijk}+u_{j}^{\left(S\right)}+v_{k}^{\left(S\right)}. \end{array}$$

Here, the same covariates (*X*) from the first stage (2.2) enter the second-stage model. The random intercepts $u_{j}^{\left(S\right)}$ and $v_{k}^{\left(S\right)}$ mirror the structure of the first stage but are estimated separately; the superscripts (T) and (S) distinguish the two stages. The coefficient $\beta_{T}$ represents the causal effect of treatment on the log-hazard of the outcome, while δ captures the adjustment for unmeasured confounding through the first-stage residual.

### 2.3 Diagnosing first-stage strength in nonlinear mixed-effects models

In linear first-stage models, weak-instrument diagnostics are based on the partial *F*-statistic testing whether the instrument predicts treatment $H_{0}:\alpha_{1}=0$ . With a single instrument, the partial *F* equals the square of the *t*-statistic on the instrument coefficient, and the conventional “strong IV” rule of thumb is F≳10 .^22^ Software packages like ivreg,^24^ fixest,^25^ and plm^26^ provide built-in tests for linear models.

When the treatment variable is binary, requiring a logistic first-stage model, no direct analog to the *F*-statistic exists. Recommended alternatives include the Wald statistic for the instrument coefficient or likelihood ratio tests.^27^ However, no standard R packages currently provide weak-instrument diagnostics for logistic mixed-effects first-stage models. Of the available alternatives, the Wald statistic is the most direct analog to the *F*-statistic.

We can test the same null $H_{0}:\alpha_{1}=0$ with a Wald statistic. Let ${\hat{\alpha }}_{1}$ be the estimated IV coefficient and $\mathrm{SE}({\hat{\alpha }}_{1})$ its standard error; define

$$\begin{array}{r} z_{\mathrm{IV}}=\frac{{\hat{\alpha }}_{1}}{\mathrm{SE}({\hat{\alpha }}_{1})},\;W=z_{\mathrm{IV}}^{\;2}. \end{array}$$

Under $H_{0}$ , *W* follows a $\chi_{1}^{2}$ distribution, from which the *p*-value can be obtained as

$$\begin{array}{r} p_{W}=1-F_{\chi_{1}^{2}}(W), \end{array}$$

where $F_{\chi_{1}^{2}}$ is the cumulative distribution function of the chi-squared distribution with one degree of freedom. We report *W* (df =1 ) as the first-stage relevance diagnostic for the logistic mixed model. However, the operating characteristics of this *W* diagnostic in IPDMA settings are not well-documented. Our simulations therefore systematically vary design knobs that affect instrument strength (instrument–exposure association strength and cluster-level instrument–exposure allocation patterns), and then stratify 2SRI performance metrics by the realized first-stage Wald $\chi^{2}$ . This links a quantity analysts can compute in practice to expected bias, coverage, and precision in mixed-effects 2SRI.

### 2.4 Hypotheses

Based on the theoretical framework and existing literature, we propose the following hypotheses:

1. Under weak confounding, mixed-effects 2SRI will not produce improved causal estimation over mixed-effects Naïve Cox.
2. Under moderate to strong confounding, mixed-effects 2SRI will improve coverage of the true effect estimate.
3. Under moderate to strong confounding, relative bias will be lower in mixed-effects 2SRI.
4. Under weak to moderate confounding, mixed-effects 2SRI will have higher variance and worse MSE than naïve survival.

## 3 Simulation studies

We follow the ADEMP framework^28^ to design and report our simulation study. The study is motivated by real-world pooled infectious disease datasets held by ReCoDID^29^ and IDDO.^30^

### 3.1 Aim

The primary aim of this study is to evaluate the performance of mixed-effects 2SRI model, relative to a mixed-effects naïve Cox proportional hazards model, and whether it yields valid estimates of the treatment log-hazard effect in a one-step IPDMA with two random effects under varying levels of unmeasured confounding.

The secondary aim is to characterize the relationship between first-stage Wald statistics and 2SRI performance across simulation scenarios, and explore whether approximate regions of Wald values correspond to acceptable levels of bias and inference. In order to do this, we systematically vary instrument–exposure association levels and instrument–exposure prevalence patterns across studies while holding the IPDMA structure fixed.

### 3.2 Data-generating mechanisms

We generate data from parametric models anchored to the sizes and structure of a pooled Ebola Virus Disease dataset held by IDDO.^31^ We fix the number of studies at 10, with study-specific sample sizes matching those observed in the Ebola dataset (100, 148, 111, 738, 1096, 185, 1230, 214, 90, 445).^31^ Sample sizes are fixed to ensure external relevance to infectious disease outbreak contexts, where studies are generally small, and computational feasibility. While sample sizes are fixed, the number of events arises from the competing risks process.

Treatment assignment is generated from a logistic regression model, including instrument assignment, unmeasured confounding, and covariates. Survival times are generated from a Weibull model with log-hazard determined by treatment, covariates, unmeasured confounding, and random effects for study and calendar month. In Ebola and similar severe infectious diseases, patients typically die or are discharged within 30 days of admission. Thus, our Weibull parameters reflect this short survival window typical of real-world outbreaks. The true treatment effect is set to log-hazard =−0.4 .

We vary three design factors in a fully factorial grid:

- *Instrument assignment pattern* (three levels): balanced (50%), moderately unbalanced (70%–30%), and a distribution based on a real-world Ebola treatment distribution (76%, 97%, 98%, 5%, 77%, 5%, 52%, 10%, 29%, 15%).^31^
- *Instrument–treatment association strength* (four levels, log-odds scale): $iv_{\text{strength}}\in \{0.3,0.5,0.8,1.0\}$
- *Unmeasured confounding* (four levels, affecting both treatment and outcome):
  - Weak: $(\beta_{U}^{\left(t\right)},\beta_{U}^{\left(d\right)})=(0.5,0.5).$
  - Moderate: (1.0,1.0).
  - Strong: (1.5,1.5).
  - Very strong: (2.0,2.0).

#### 3.2.1 Parameterization of unmeasured confounding

Let *U* denote an unobserved patient-level factor that affects both treatment assignment and the outcome. In our generator, we fix $U∼N(0,\;0.5^{2})$ and vary the coefficients of *U* in the treatment and outcome models, $\left(\beta_{U}^{\left(t\right)},\beta_{U}^{\left(d\right)}\right)$ , to control confounding intensity. With sd(U)=0.5 fixed, our levels therefore target per-standard-deviation shifts of size $\beta_{U}\times 0.5$ on the logit (first stage) and log-hazard (second stage) scales. These tiers mirror the low/medium/high confounding regimes obtained by varying sd(U) in prior work,^32^ while keeping the implementation simple and transparent through the coefficients.

#### 3.2.2 Instrument–exposure association (first-stage design strength)

We manipulate the design-time instrument–exposure association via the coefficient on the instrument in the first-stage logistic mixed model:

$$\begin{array}{r} \mathrm{logit}\mathrm{Pr}(T=1)=\alpha_{0}+\underset{\text{design strength}}{\underbrace{\alpha_{1}Z}}+\text{covariates}+u_{\text{study}}+v_{\text{time}}. \end{array}$$

Varying $\alpha_{1}$ directly controls the odds ratio $\exp(\alpha_{1})$ for *Z* and thus the induced shift in treatment propensity (holding other terms fixed). This follows the standard approach used in simulation studies that tune instrument relevance through the first-stage slope on *Z* (e.g., $\alpha_{1}\in \{1,2,3\}$ ^32^). We use a slightly finer grid to improve resolution:

$$\begin{array}{r} \alpha_{1}\in \{0.3,\;0.5,\;0.8,\;1.0\}. \end{array}$$

Realized first-stage diagnostics (e.g., Wald $\chi^{2}$ ) are reported descriptively but are not used to set design strength.

#### 3.2.3 Instrument allocation patterns across studies

We generate a binary instrument $Z_{ij}∼\mathrm{Bernoulli}(p_{j})$ with study-specific probabilities $p_{j}$ . Patterns include balanced ( $p_{j}=0.50$ ), moderately unbalanced ( 0.70/0.30 ), and a heterogeneous empirical pattern derived from treatment assignment patterns in the pooled Ebola dataset.^31^ This construction is consistent with preference-style instruments in the literature, which use either a single overall prevalence (e.g., P(Z=1)≈0.7 )^32^ or cluster-specific propensities spanning a realistic range (e.g., hospital preferences between roughly 17% and 58%).^33^ In our design, $\alpha_{1}$ governs the instrument signal, while $\left\{p_{j}\right\}$ governs the overlap and information; we vary them orthogonally.

*Treatment assignment*: Treatment assignment follows a logistic model

$$\begin{array}{r} \mathrm{logit}\mathrm{Pr}(T_{ijk}=1)=-0.2+\text{iv\_strength}\cdot Z_{ijk}+\beta_{U}^{\left(T\right)}\;U_{i}+\beta_{\text{me}}\;\varepsilon_{i}^{\left(\text{me}\right)}+u_{j}^{\left(T\right)}+v_{k}^{\left(T\right)}, \end{array}$$

with $Z_{ijk}∼\mathrm{Bernoulli}(p_{j})$ given the instrument assignment pattern across studies, $U_{i}∼N(0,0.5^{2})$ , $u_{j}^{\left(T\right)}∼\mathrm{Uniform}(-0.1,0.1)$ , $v_{k}^{\left(T\right)}∼N(0,0.15^{2})$ , and $\beta_{\text{me}}=0.1$ . Here, $\varepsilon_{i}^{\left(\mathrm{me}\right)}$ is a mean-zero individual-level noise (measurement-error) term entering the first stage via $\beta_{\mathrm{me}}\;\varepsilon_{i}^{\left(\mathrm{me}\right)}$ .

*Outcomes*: In severe infectious disease outbreaks such as Ebola, survival times are short, with discharge or death typically occurring under 30 days, so the baseline hazard operates over a narrow window. We define the cause-specific hazards for death and discharge by $h_{ijk}^{\text{death}}(t)$ and $h_{ijk}^{\text{disch}}(t)$ , respectively, where *i* indexes individuals, *j* studies, and *k* time. The corresponding log-hazard models are

$$\begin{array}{r} \begin{array}{rl} \log h_{ijk}^{\text{death}}(t) & =-4.2+u_{j}^{\left(S\right)}+v_{k}^{\left(S\right)}+\beta_{T}\;T_{ijk}+\beta_{\text{age}}\;{\text{age}}_{i}+\beta_{\text{sex}}\;1\{{\text{Male}}_{i}\}+\beta_{U}^{\left(S\right)}\;U_{i},\\ \log h_{ijk}^{\text{disch}}(t) & =-1.1-u_{j}^{\left(S\right)}-v_{k}^{\left(S\right)}-\beta_{T}\;T_{ijk}-\beta_{\text{age}}\;{\text{age}}_{i}-\beta_{\text{sex}}\;1\{{\text{Male}}_{i}\}-\beta_{U}^{\left(S\right)}\;U_{i}, \end{array} \end{array}$$

where $u_{j}^{\left(S\right)}∼\mathrm{Uniform}(-0.2,0.2)$ , $v_{k}^{\left(S\right)}∼N(0,0.15^{2})$ , $\beta_{T}=-0.4$ , $\beta_{\text{age}}=0.005$ , $\beta_{\text{sex}}=0.1$ , and $\left(\beta_{U}^{\left(T\right)},\beta_{U}^{\left(S\right)})\in \{(0.5,0.5),(1.0,1.0),(1.5,1.5),(2.0,2.0)\right\}$ by confounding level.

*Random effects structure*: Study-level random effects were drawn from Uniform distributions to induce bounded heterogeneity, while calendar-time effects were drawn from Normal distributions to allow occasional larger deviations. These choices ensure simulation stability across the factorial grid. This structure is mirrored in the analysis models, which include random intercepts for study and admission month.

Times are drawn

$$\begin{array}{r} T_{i}^{\text{death}}∼\mathrm{Weibull}(1.2,\;0.45/h_{ijk}^{\text{death}}(t)),\;T_{i}^{\text{disch}}∼\mathrm{Weibull}(6.0,\;3.0/h_{ijk}^{\text{disch}}(t)), \end{array}$$

then $Y_{i}=\min\{T_{i}^{\text{death}},T_{i}^{\text{disch}}\}$ and $\Delta_{i}=1\{T_{i}^{\text{death}}\leq T_{i}^{\text{disch}}\}$ .

### 3.3 Estimand

The primary estimand is the cluster-conditional treatment log-hazard effect in the structural hazard model, with true value $\theta =\beta_{T}=-0.4$ . We evaluate whether each estimator (naïve mixed-effects Cox and mixed-effects 2SRI) recovers θ under varying design factors.

### 3.4 Methods

For each simulated dataset, we estimate the treatment effect using (i) a naïve mixed-effects Cox model (fit via coxme ^34^) adjusting for age and sex with random effects for study and time and (ii) mixed-effects 2SRI, with a first-stage logistic mixed-effects model (fit via lme4 ^35^) adjusting for age and sex with random effects for study and time, and a second-stage Cox mixed-effects model (fit via coxme) with residual inclusion, adjusting for age and sex with random effects for study and time. Analyses are run in R (RStudio 2024.12.1+563, “Kousa Dogwood”),^36^ with R code available on GitHub and archived on Zenodo: https://doi.org/10.5281/zenodo.20022540.

### 3.5 Performance measures and Monte Carlo precision

For the primary aim, we evaluate bias, coverage, empirical SE, and MSE; for the secondary aim, we examine how these same metrics vary across realized first-stage Wald $\chi^{2}$ values.

*Terminology*: We distinguish (i) the *model-based standard error* ${\hat{\mathrm{SE}}}_{i}$ returned by each fitted model in replication *i* (used to form Wald CIs and compute coverage), from (ii) the *empirical variability across replications*, $s_{\hat{\theta }}=\sqrt{\frac{1}{n-1}\sum_{i=1}^{n}({\hat{\theta }}_{i}-\overset{―}{\hat{\theta }}{)}^{2}}$ , which we label “Empirical SE” in figures.

*Relative bias (%):*

(3.1) $$\begin{array}{r} \mathrm{RB}=100\times \frac{\left|\overset{―}{\hat{\theta }}-\theta \right|}{\left|\theta \right|}. \end{array}$$

*Coverage (95% CI):*

(3.2) $$\begin{array}{r} \hat{p}\;=\;\frac{1}{n}\sum_{i=1}^{n}1\;\left\{\theta \in \left[{\hat{\theta }}_{i}\pm 1.96\;{\hat{\mathrm{SE}}}_{i}\right]\right\}, \end{array}$$

where ${\hat{\mathrm{SE}}}_{i}$ is the model-based standard error from replication *i*.

*Empirical SD of* $\hat{\theta }$ *(“Empirical SE” in figures):*

(3.3) $$\begin{array}{r} \hat{\mathrm{SD}}(\hat{\theta })\;=\;s_{\hat{\theta }}=\sqrt{\frac{1}{n-1}\sum_{i=1}^{n}\;({\hat{\theta }}_{i}-\overset{―}{\hat{\theta }}{)}^{2}}. \end{array}$$

*Mean squared error (MSE):*

(3.4) $$\begin{array}{r} \mathrm{MSE}=\frac{1}{n}\sum_{i=1}^{n}\;({\hat{\theta }}_{i}-\theta {)}^{2}. \end{array}$$

We also record the realized distribution of first-stage instrument strength (Wald statistic $W=z_{\text{IV}}^{\;2}$ ).

#### 3.5.1 Smoothing and uncertainty displays

Smoothing was used only in exploratory, pre-analysis plots: (i) LOESS trend lines in $\hat{\beta }$ versus the model-based $\mathrm{SE}(\hat{\beta })$ scatterplots; (ii) quadratic least-squares fits in the instrument-to-Wald “bridge” figures; and (iii) density overlays. We did not use GAMs, binomial smoothers, or ribbons. Main performance figures (relative bias, empirical SD, MSE, and coverage) display cell means with 95% Monte Carlo (MC) confidence intervals. Figure labels use the terminology defined above.

#### 3.5.2 Monte Carlo precision and choice of 1,000 replications per scenario

We quantify MC uncertainty for each scenario-level summary using analytic MC standard errors (MC-SEs) that scale as $n^{-1/2}$ , where *n* is the number of replications.

*Proportions (coverage)*: With n=1,000 and coverage near 95%, the binomial MC-SE is

$$\begin{array}{r} \sqrt{\frac{0.95\;\times \;0.05}{1000}}\approx 0.0069, \end{array}$$

yielding a 95% MC interval half-width of 1.96×0.0069≈1.35 percentage points. The worst case for proportions ( p=0.5 ) gives MC-SE =0.0158 (half-width ≈3.10 percentage points).

*Continuous summaries (bias)*: For the scenario-level bias, $\overset{―}{\hat{\theta }}-\theta$ , the MC-SE is

$$\begin{array}{r} \mathrm{MC}\text{-}\mathrm{SE}(\overset{―}{\hat{\theta }}-\theta )=\frac{s_{\hat{\theta }}}{\sqrt{n}}, \end{array}$$

where $s_{\hat{\theta }}$ is the across-replication standard deviation of $\hat{\theta }$ . In our runs $s_{\hat{\theta }}\in [0.15,\;0.25]$ , so at n=1,000, the MC-SE is ≈0.005 – 0.008 (about 1%–2% of |θ|=0.4 ).

*Empirical SD and MSE*: We use standard analytic formulas for the MC uncertainty of the across-replication SD and of the MSE; these appear as error bars on the forest plots.

*Rationale for* n=1,000 : MC uncertainty decreases as $1/\sqrt{n}$ . At 1,000 replications, the 95% MC interval for coverage is approximately ±1.35 percentage points; increasing to 2,000 and 4,000 would reduce this to ∼±0.95 and ∼±0.68 percentage points, respectively, at 2 × and 4 × the computational cost. We therefore adopt 1,000 replications per scenario as a balanced choice that yields decision-grade precision without disproportionate computational cost.

## 4 Results

Results are organized per ADEMP guidelines,^28^ 4.1, visualizations of results by DGMs to check for patterns, missing values, and outliers; 4.2, main performance results comparing mixed-effects Cox survival model (hereafter referred to as Naïve) to mixed-effects 2SRI (hereafter referred to as 2SRI), stratified by realized IV strength quintiles and unmeasured confounding levels; and 4.3, relationship between design parameters and realized IV strength (Wald $\chi^{2}$ , df=1) distributions. Comprehensive visualization figures broken down by individual DGM factors are provided in Figures A1–A6 in the Supplementary Material.

### 4.1 Visualization of results

All 48 simulation scenarios were completed successfully with 1,000 replications each, yielding no missing values.

**Figure 2:**
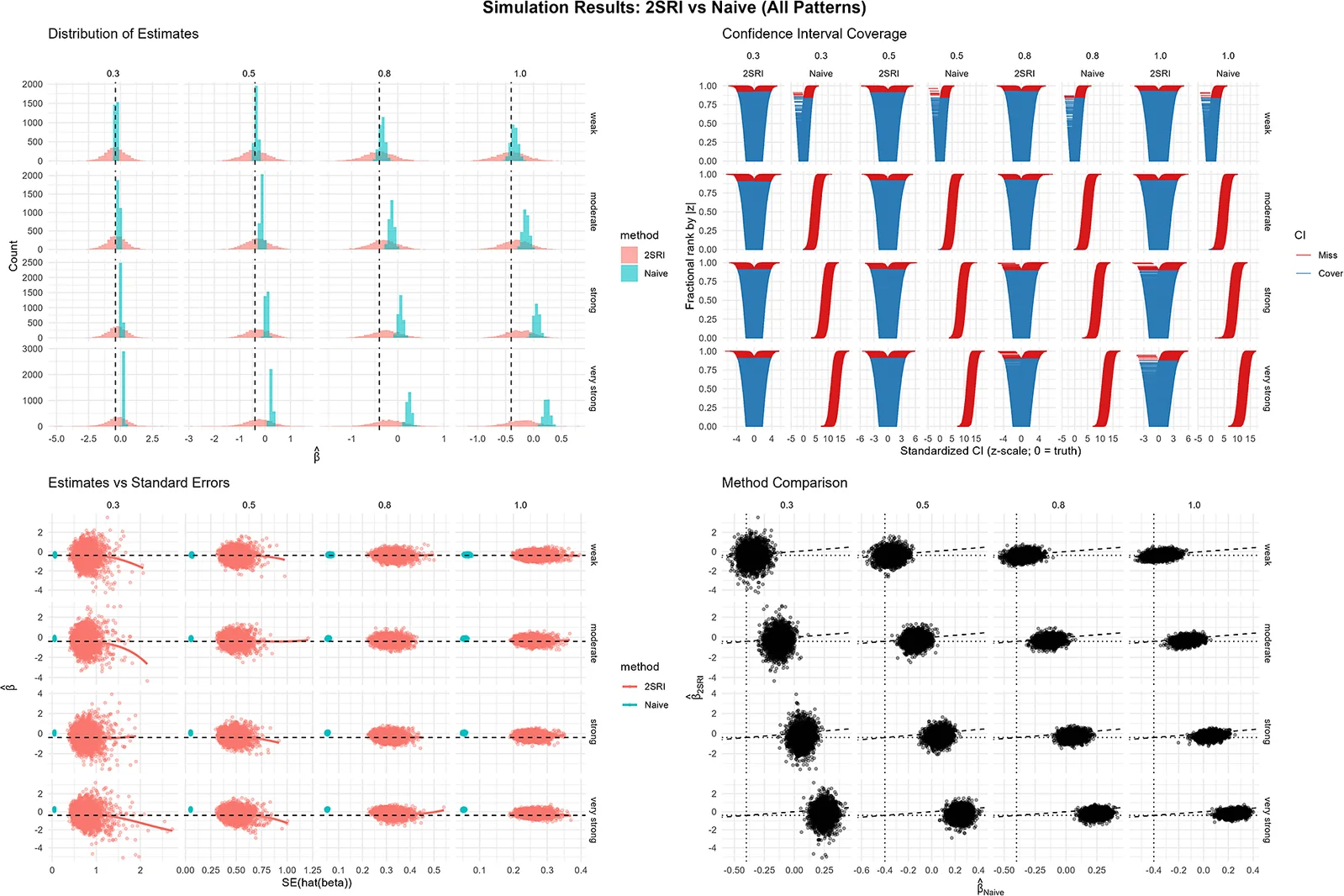
Simulation results comparing 2SRI and naïve estimation methods. Top left: Distribution of parameter estimates. Top right: Confidence-interval coverage. Bottom left: Estimates versus standard errors. Bottom right: Method-to-method comparison. Results are stratified by IV–exposure strength (0.3, 0.5, 0.8, 1.0; top facets) and unmeasured confounding (weak, moderate, strong, very strong; right facets). Across all IV strengths and confounding levels, 2SRI remains centered near the true effect and attains near-nominal coverage, with model-based SE s shrinking as instrument strength increases; in contrast, the naïve estimator shows small SE s but increasing bias and near-zero coverage as confounding rises. In the method-comparison panel, divergence from the diagonal equality line ( y=x ) widens with confounding—indicating growing disagreement driven by naïve bias—while points remain close to the horizontal truth line at θ=−0.4 . Figure 2 Long description.

Figure 2 summarizes key diagnostics: distributions of $\hat{\beta }$ and model-based $\text{SE}(\hat{\beta })$ , confidence interval coverage, $\hat{\beta }$ versus model-based $\text{SE}(\hat{\beta })$ with truth lines, and method-comparison scatter plots, each stratified by confounding level and IV–exposure association strength. The diagnostics show expected weak-instrument pathology for 2SRI (rare but extreme outliers and inflated SEs at lower IV strengths), with SEs shrinking as strength increases. Coverage for 2SRI is generally high, while the naïve estimator’s coverage approaches 0% under moderate to very strong confounding. Comprehensive visualizations are provided in Figures A1–A6 in the Supplementary Material, including detailed analyses by each DGM factor for univariate distributions, method comparisons, and coverage assessments.

### 4.2 Main performance results

#### 4.2.1 Relative bias (%)

**Figure 3:**
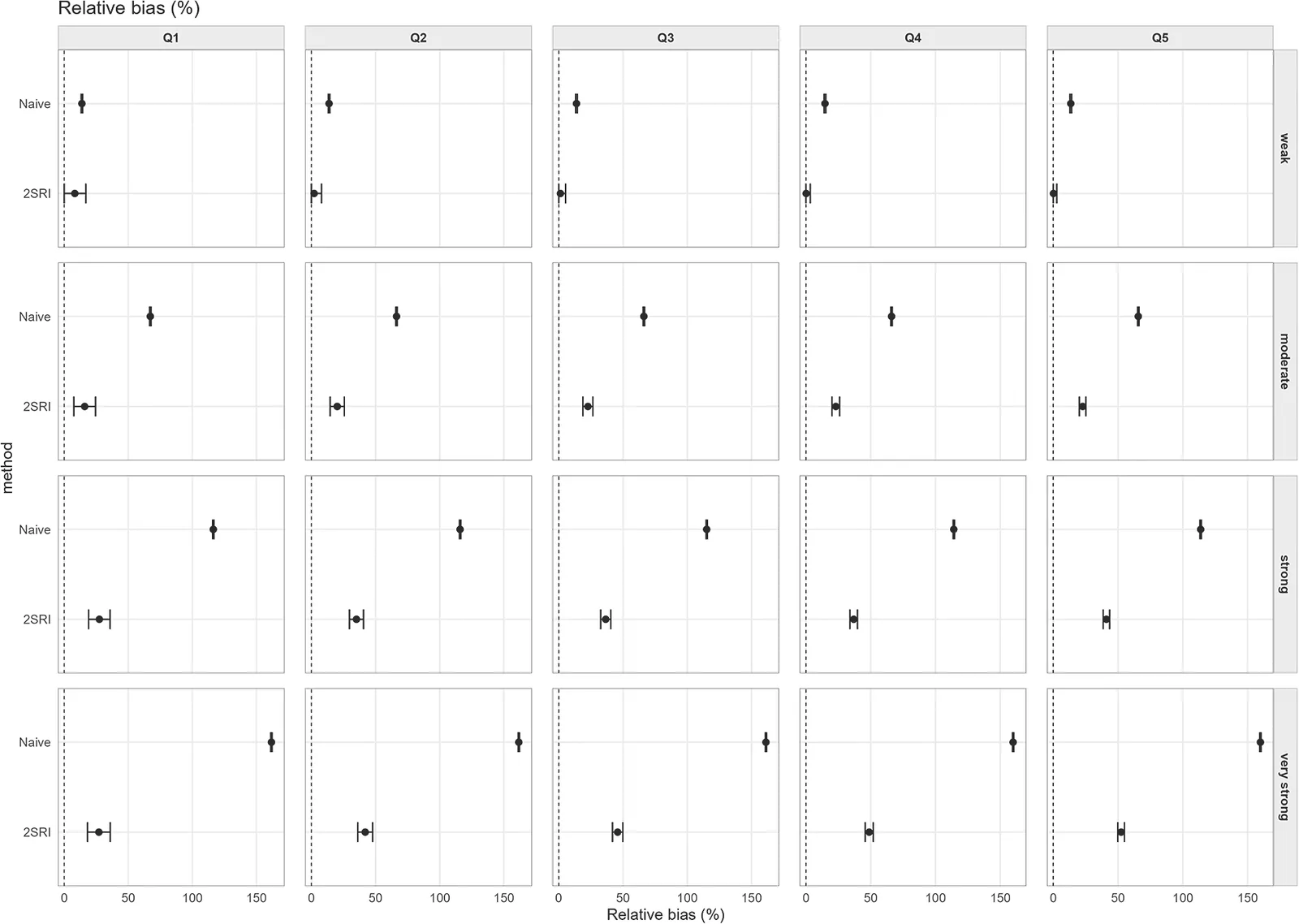
Relative bias (%) by unmeasured confounding (*right facets*) and realized IV strength (Wald $\chi^{2}$ quintiles Q1–Q5; *top facets*). Points show cell means for each method (2SRI, naïve) with vertical bars showing 95% Monte Carlo CIs. Naïve relative bias grows rapidly with stronger confounding and remains large across quintiles, whereas 2SRI exhibits substantially lower bias that is fairly stable across quintiles. Figure 3 Long description.

Relative bias is defined in (3.1). Figure 3 displays percent relative bias across unmeasured confounding levels and realized Wald $\chi^{2}$ quintiles (Q1–Q5, binned locally within each confounding level). The naïve model’s relative bias rises sharply with unmeasured confounding, exceeding 100% under strong confounding. 2SRI shows smaller bias than naïve, starting from the moderate unmeasured confounding level.

#### 4.2.2 Coverage (95% CI)

**Figure 4:**
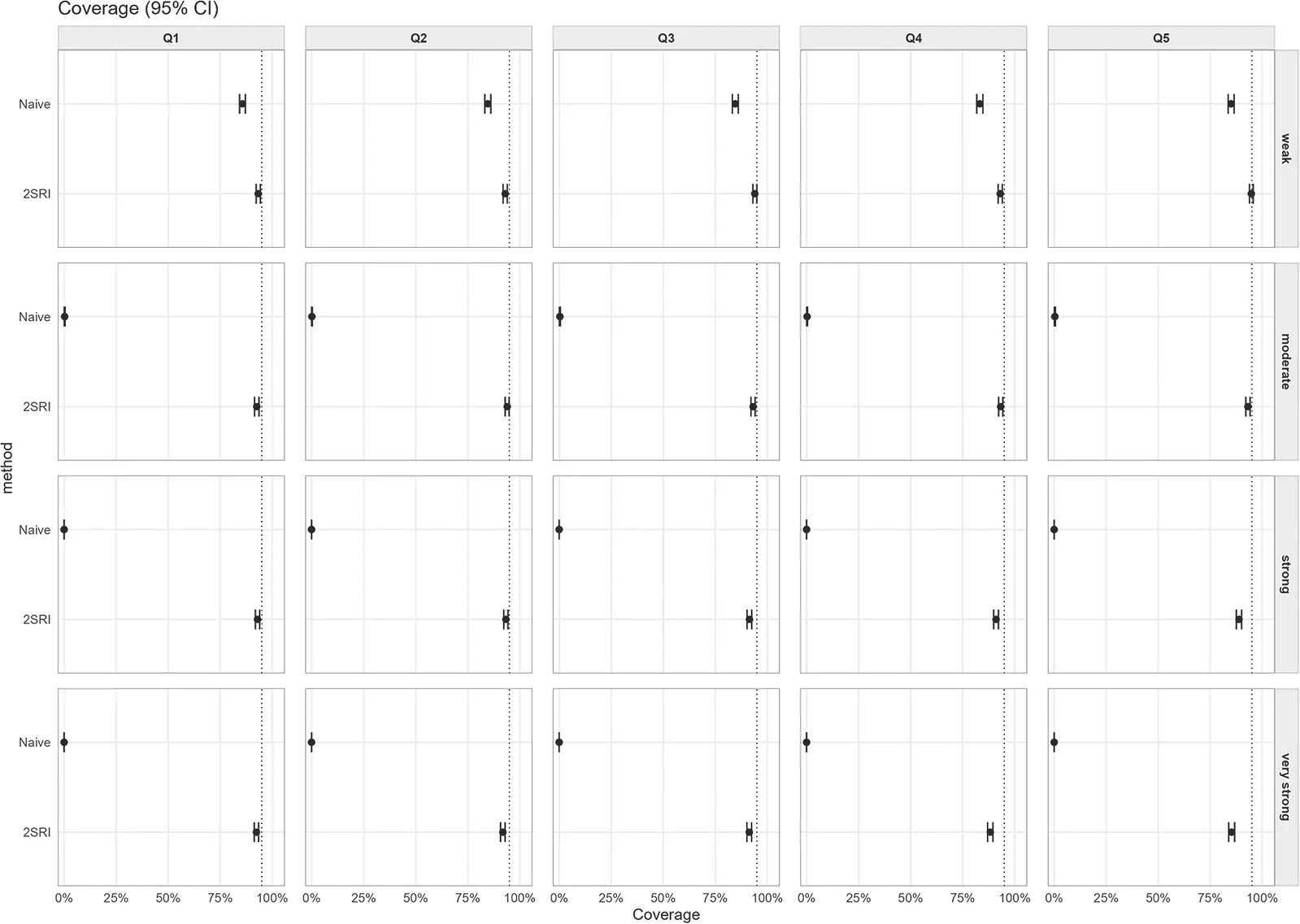
Coverage of nominal 95% CIs by unmeasured confounding (*right facets*) and realized IV strength (Wald $\chi^{2}$ quintiles Q1–Q5; *top facets*). Points are observed coverages with 95% Monte Carlo CIs; the vertical dashed line marks 95% target coverage. Naïve coverage deteriorates beginning at moderate confounding, whereas 2SRI approaches target coverage across all confounding levels and Wald $\chi^{2}$ quintiles. Figure 4 Long description.

In Figure 4, 2SRI achieves near-nominal coverage (see (3.2)) across all confounding levels and Wald $\chi^{2}$ strength quintiles (Q1–Q5, binned locally within each confounding level). By contrast, naïve estimates collapsed as confounding increased, dropping below 5% under strong and very strong confounding, and variation across bins is minimal.

#### 4.2.3 Empirical standard error

**Figure 5:**
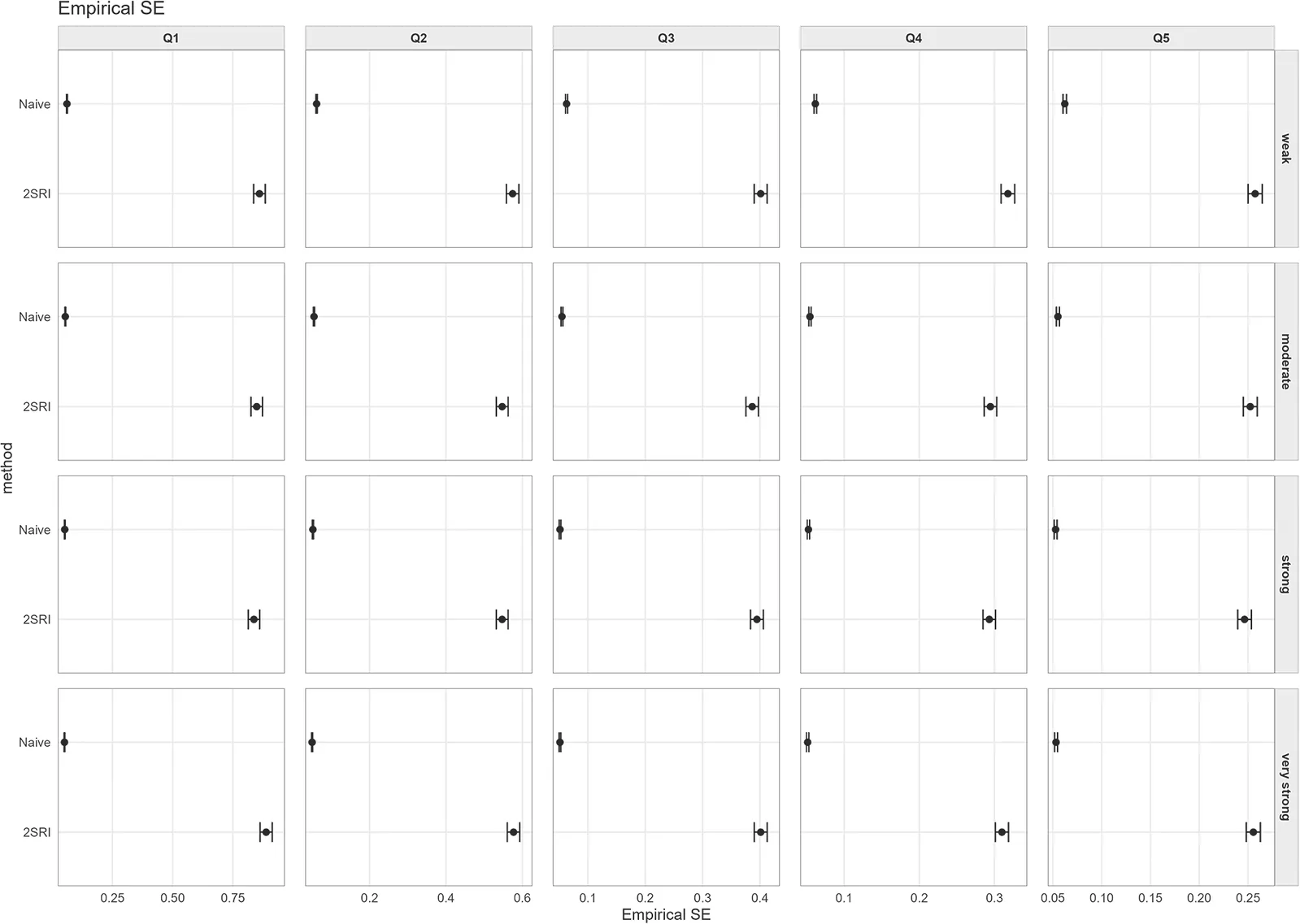
Empirical standard error of $\hat{\beta }$ by unmeasured confounding (*right facets*) and realized IV strength (Wald $\chi^{2}$ quintiles Q1–Q5; *top facets*). Points are cell means with vertical bars showing 95% MC CIs. 2SRI has inflated SEs with weak instruments (Q1 and Q2) and smaller SEs as IV strength increases (Q4 and Q5), while naïve SEs remain small across all confounding levels. Figure 5 Long description.

The precision trade-off between naïve survival and 2SRI is shown in Figure 5, which plots the empirical SE. The naïve estimator has uniformly small SEs (approximately 0.05–0.06), consistent with overconfident inference when biased. For 2SRI, SE is markedly inflated under weak instruments (Q1, approximately 0.8–0.9) and remains elevated in Q2 (approximately 0.5–0.6), then declines to approximately 0.4 in Q3, and stabilizes around 0.35 or below by Q4 and Q5. Within each Wald quintile, 2SRI SE is fairly constant across confounding levels.

#### 4.2.4 Mean squared error

**Figure 6:**
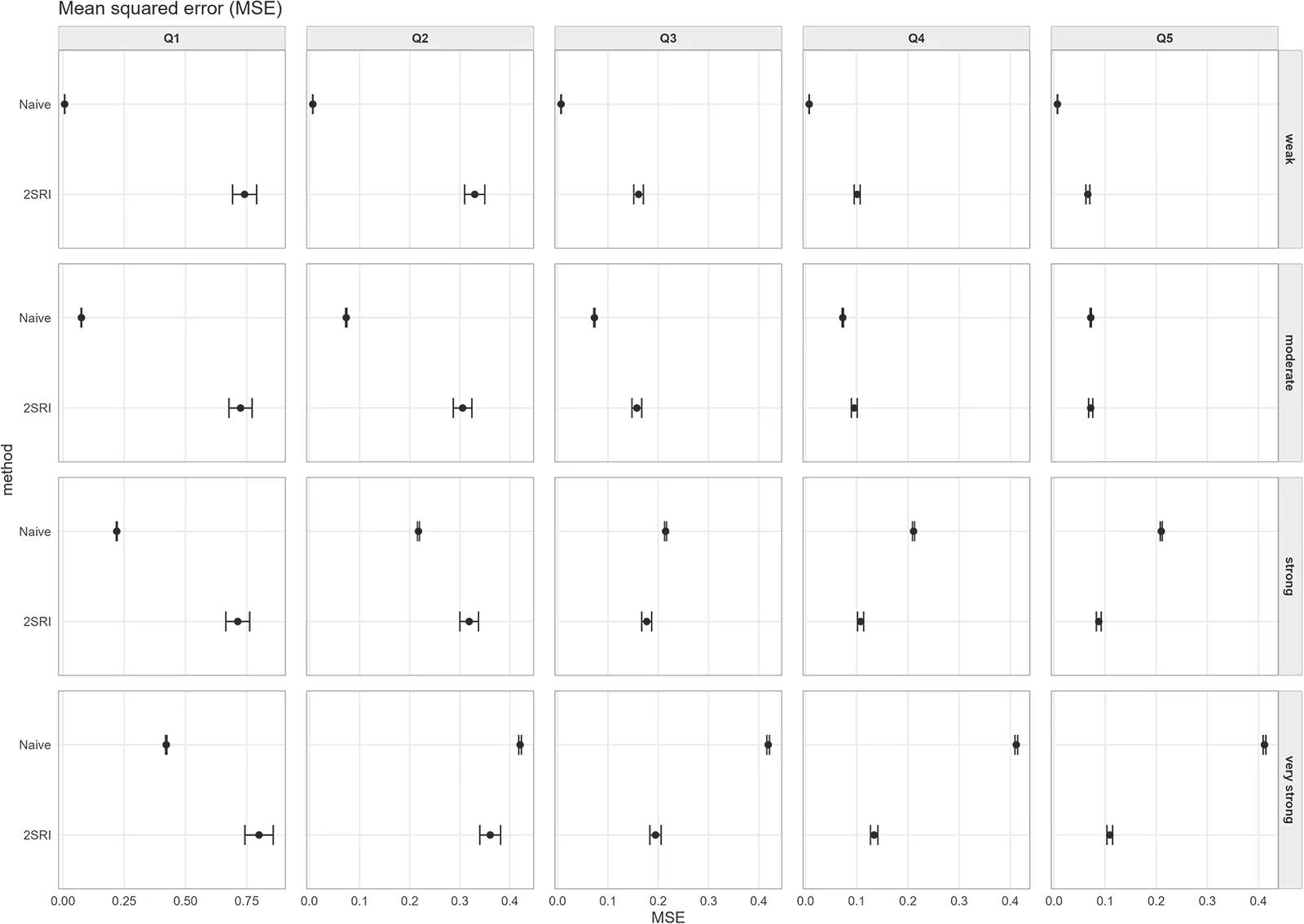
Mean squared error (MSE) by unmeasured confounding (*right facets*) and realized IV strength (Wald $\chi^{2}$ quintiles Q1–Q5; *top facets*). Points are cell means with vertical bars for 95% MC CIs. Naïve MSE increases with confounding. 2SRI’s MSE is variance-dominated under weak instruments (Q1 and Q2) but improves as realized IV strength and confounding increases. Under strong or very strong confounding, 2SRI attains lower MSE than naïve once the instrument is at least moderate. Figure 6 Long description.

MSE combines bias and variance (see (3.4); Figure 6). With weak and moderate unmeasured confounding, the naïve estimator has lower MSE than 2SRI across all instrument-strength quintiles. Under strong confounding, 2SRI has lower MSE across Q3–Q5. With very strong confounding, the naïve estimator dominates for Q1, but 2SRI attains lower MSE from Q2 to Q5.

#### 4.2.5 Interpreting performance through realized instrument strength (secondary aim)

**Figure 7:**
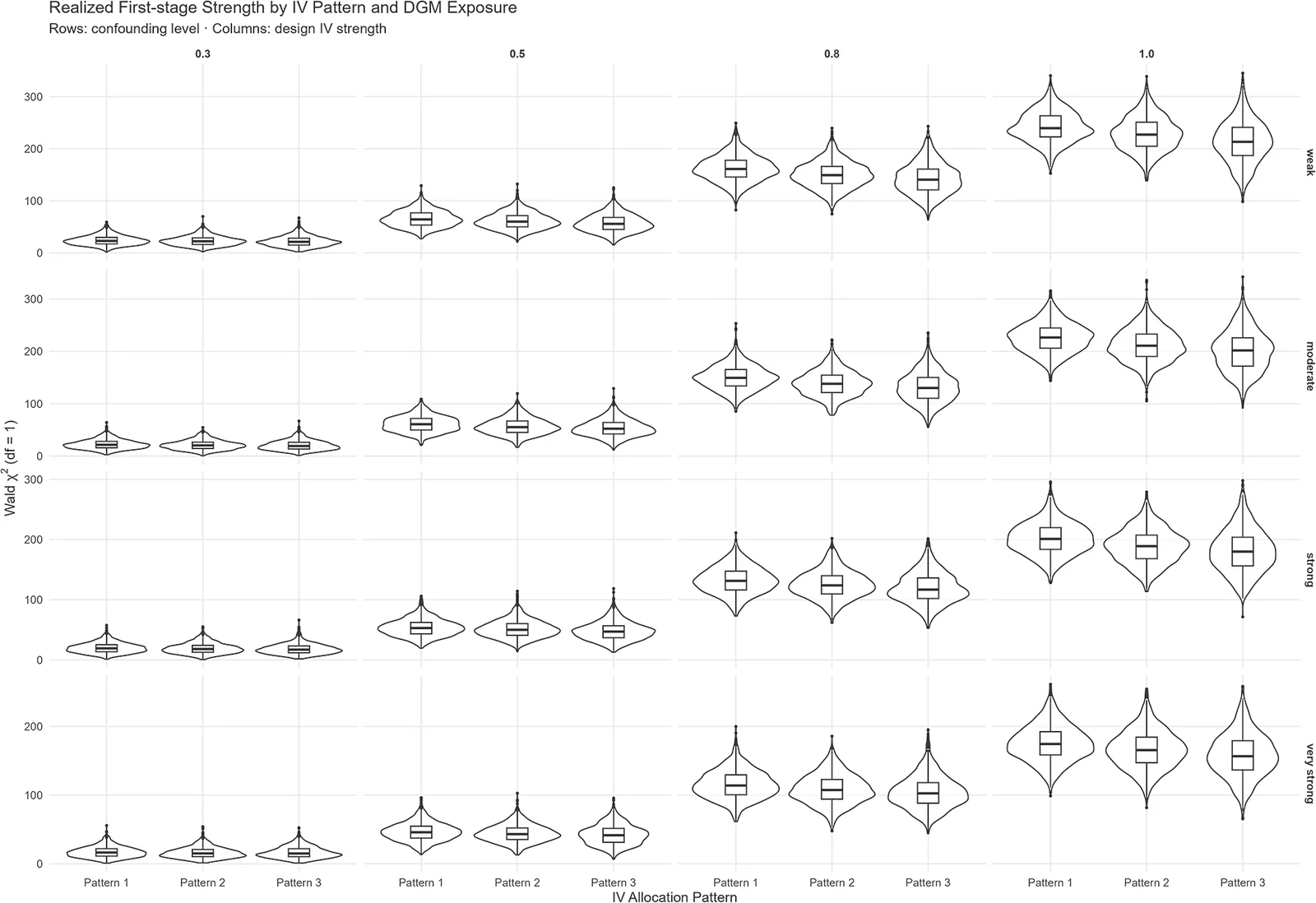
Realized IV strength (Wald $\chi^{2}$ ; df=1) by IV allocation scheme. Violin/box plots are shown across IV–exposure strengths (0.3–1.0; *top facets*) and unmeasured confounding levels (weak to very strong; *right facets*). Instrument-strength distributions widen with increasing IV–exposure correlation, while overall shapes remain similar across allocation patterns. Figure 7 Long description.

*How design generates Wald chi-square statistics*: Figure A7 in the Supplementary Material shows how the two IV-related design knobs map to realized IV strength (Wald $\chi^{2}$ , df=1): as IV–exposure strength on the *x*-axis increases from 0.3 to 1.0 , realized Wald rises monotonically and convexly. Across all confounding levels, allocation Pattern 1 yields the largest Wald $\chi^{2}$ values, followed by Patterns 2 and 3, although the effect of the IV–exposure allocation pattern was comparatively minor compared to the IV–exposure association level, and differences between patterns decreased as confounding increased. The violin panels in Figure 7 and histograms in Figure A8 in the Supplementary Material confirm these distributional shifts: moving from 0.3 to 1.0 shifts the entire Wald $\chi^{2}$ distribution rightward and increases its right tail; pattern-specific medians separate consistently with the same ordering. While confounding level substantially affects estimator performance, it also reduces realized Wald $\chi^{2}$ values—most notably at higher IV strengths, where strong confounding can reduce Wald values by approximately 25% (from 230–240 to 170–180 at IV strength 1.0). As concrete visual anchors: medians around IV–exposure 0.3 sit near the W≈20 – 50 , 0.5 sit near the W≈50 – 80 range, around 0.8 near W≈100 – 160 , and 1.0 near W≈160 – 240 (see Figure 7, and Figures A7 and A8 in the Supplementary Material).

*Linking Wald to performance*: Because applied analysts observe the realized first stage (not the DGM knobs), the main performance figures—relative bias, coverage, empirical SE, and MSE (Figures 3–6)—should be read through the value of *W*: panels corresponding to lower realized Wald $\chi^{2}$ exhibit classic weak-IV behavior for 2SRI (larger, more variable bias; sub-nominal coverage; inflated SEs), whereas panels with higher realized Wald $\chi^{2}$ show reduced 2SRI bias, near-nominal coverage, and stabilized SEs. In our simulations, 2SRI performance was unstable when Wald $\chi^{2}$ values clustered below 50, improved between 100 and 150, and showed improved MSE over naïve once values exceeded 150–200. These are not universal thresholds but illustrate performance ranges mapped to realized Wald $\chi^{2}$ values for our specific study structure.

To complement Figures 3–7, and Figures A7 and A8 in the Supplementary Material, Tables A1–A5 in the Supplementary Material report scenario-level summaries. Table A1 in the Supplementary Material aggregates relative bias, empirical SE, MSE, and coverage by design-stage IV–exposure strength, while Tables A2–A5 in the Supplementary Material cross-tabulate the same metrics by unmeasured confounding level and IV design knobs (allocation pattern × IV–exposure strength).

### 4.3 Sensitivity to exclusion and independence violations

The primary simulations varied instrument strength to examine performance under weak relevance. To complete the assessment across all three IV assumptions, we conducted additional simulations introducing violations of the exclusion restriction (direct instrument–outcome effect, γ=0.08 ) and independence assumption (instrument–confounder correlation, α=0.2 ), holding IV strength at 0.8 and instrument allocation balanced. Under both strong and very strong confounding, these violations substantially degraded 2SRI performance (Figures B1 and B2 and Table B1 in the Supplementary Material). Exclusion violations increased relative bias from approximately 38% to 105% under strong confounding, with coverage falling from 90% to 70%. Independence violations had a similar impact, increasing bias to 99% with coverage around 65%. Together with the relevance findings in Section 4.2, these results demonstrate that 2SRI performance depends critically on all three identifying assumptions.

## 5 Discussion

To the best of our knowledge, this article presents one of the first systematic adaptation and evaluation of a mixed-effects 2SRI method for IPDMAs. To evaluate performance, we used a full factorial design varying (i) the magnitude of unmeasured confounding (four levels), (ii) the IV–exposure association (four levels), and (iii) cross-study instrument allocation ( $p_{j}\in \{50/50,\;70/30,\;\text{empirical}\}$ ), yielding 48 scenarios while holding the IPDMA architecture (10 studies with fixed sizes) constant. Because the classical *F*-statistic is not defined for mixed-effects logistic first-stage models, we use the Wald $\chi^{2}$ (df=1) for the instrument coefficient as a practical diagnostic of realized first-stage strength and evaluate performance metrics conditional on this statistic (quintiles Q1–Q5).

### 5.1 Main findings

Under weak unmeasured confounding, the naïve model generally dominates on bias and MSE. With moderate confounding, 2SRI improves relative bias and coverage but pays a variance cost in MSE. When confounding is strong and valid instruments are moderate to strong (Q3–Q5), 2SRI outperforms the naïve model across all performance metrics.

However, 2SRI is not universally preferable. When the relevance assumption is weakly satisfied, the variance penalty renders 2SRI inferior on MSE grounds despite reduced bias. When the exclusion restriction and independence assumptions are violated under strong confounding, 2SRI loses its MSE advantage and can produce comparable or greater bias than naïve estimation (Appendix B of the Supplementary Material). Under very strong confounding, 2SRI retains lower MSE than naïve even with these violations, though with substantially degraded bias and coverage.

### 5.2 Practical guidance

These results highlight that the choice between naïve estimation and IV-based adjustment reflects an inherent bias–variance trade-off. In the presence of unmeasured confounding, naïve estimators may yield precise but biased estimates and potentially incorrect conclusions. Conversely, IV-based approaches, such as 2SRI, may reduce bias when the identifying assumptions hold, but at the cost of increased variance and, in some cases, inconclusive inference due to wide confidence intervals. Importantly, neither outcome is uniformly preferable: reduced bias does not guarantee more informative conclusions, and increased precision does not guarantee validity.

In this context, this study provides practical performance guides for the use of Wald $\chi^{2}$ when *F*-statistics are unavailable for logistic first-stage IV models in mixed models. Our simulations provide empirically grounded guideposts for interpreting the realized Wald $\chi^{2}$ in hierarchical survival IPDMAs (e.g., W<50 , 50 – 100 , 100 – 200 , > 200), showing how bias, variance, coverage, and MSE trade-off across these bands. In our simulations, realized *W* was driven primarily by the IV–exposure association, with smaller contributions from across-study IV exposure allocation patterns and confounding levels.

The Wald $\chi^{2}$ statistic is a test of the relevance assumption only. It provides no information about the exclusion restriction or independence assumptions. As shown in Appendix B of the Supplementary Material, 2SRI performance degrades substantially when these assumptions are violated, even with strong relevance. Analysts must therefore supplement the *W* test with theoretical justification, for example, using directed acyclic graphs and substantive knowledge of the clinical context and disease setting, to defend the plausibility of exclusion and independence before relying on 2SRI estimates.

While the Wald $\chi^{2}$ and *F*-statistic are asymptotically equivalent for a single instrument, the Staiger and Stock F>10 threshold was derived for linear 2SLS.^22^ Our simulations suggest that substantially higher Wald $\chi^{2}$ values are needed in nonlinear mixed-effects settings—an empirical finding rather than a theoretically derived result.

### 5.3 Strengths and limitations

The ADEMP-based^28^ simulation design ensured transparent evaluation. The fully-factorial design varies instrument–exposure association, across-study allocation patterns, and unmeasured confounding levels. The data-generating mechanisms reflect outbreak IPDMAs through study- and calendar-level heterogeneity, and short, ICU-like lengths of stay. We also offer a practical “bridge” from design settings to realized first-stage strength via the Wald $\chi^{2}$ , allowing readers to interpret performance figures using a quantity they can compute.

Our realized-strength bands ( W<50 , 50 – 100 , 100 – 200 , > 200) are empirically grounded guideposts, not hard thresholds. They arise from a simulation that held the IPDMA architecture fixed (numbers and sizes of studies), used a specific first-stage model (logistic mixed model with random intercepts for study and calendar month), and a Cox mixed-effects second stage, with binary instruments and the set of IV allocation patterns examined. We did not systematically vary the total sample size, the number of contributing studies, or alternative event processes and censoring schemes; nor did we explore model misspecification of the first stage or alternative frailty structures. Because Wald $\chi^{2}$ is a function of design, sample size, and heterogeneity, the quantitative boundaries that separate the quintile-like performance regimes we report may change under other settings.

Practically, we recommend that analysts report the realized first-stage Wald $\chi^{2}$ , locate their study on the performance gradients we present, and treat our ranges as decision aids to be corroborated by design-specific diagnostics (e.g., alternative first-stage specifications, overlap checks, and sensitivity analyses). Future work should map how these thresholds evolve with the number and size of studies and with alternative data-generating and modeling choices.

IV analyses face challenges in IPDMAs composed of non-randomized studies. In such settings, unmeasured confounding, treatment practices, and instrument–exposure relationships may vary across studies and over time, and it’s possible that no single instrument can be assumed to adjust for all sources of unmeasured confounding uniformly. As in any IV analysis, identification relies on the plausibility of relevance, exclusion, and independence assumptions, potentially conditional on observed covariates. However, these assumptions are inherently untestable and may hold only approximately or locally within subsets of the data. Accordingly, mixed-effects 2SRI should not be interpreted as a guarantee of causal identification in IPDMAs, but rather as a principled extension of standard IV methods whose performance depends on design, context, and assumption plausibility. Our simulation results are intended to characterize these trade-offs rather than to suggest that IV adjustment can fully resolve confounding in complex meta-analytic settings.

### 5.4 Future directions

Future research should recalibrate Wald-based guideposts across different IPDMA study sizes, numbers of studies, and event processes to establish more generalizable diagnostics. Bayesian extension of mixed-effects 2SRI may help stabilize inference under weak instruments and small study sizes, and better quantify uncertainty in hierarchical survival settings.

Our DGMs assume a common treatment effect across studies. This simplification allowed for the isolation of the effect of instrument strength and unmeasured confounding DGMs on estimator performance. In practice, treatment effects may vary across studies, and future investigations should investigate whether mixed-effects 2SRI appropriately recovers the average effect under such heterogeneity.

## 6 Conclusion

This simulation work with mixed-effects 2SRI extends IV analysis to hierarchical survival IPDMAs under common effect data-generating mechanisms. In this setting, it can outperform naïve models when realized first-stage strength and unmeasured confounding are strong.

A central contribution of this work is the use of the realized first-stage Wald $\chi^{2}$ statistic as a practical relevance diagnostic in mixed-effects logistic models where classical *F*-statistics are not available. Mapping realized Wald $\chi^{2}$ values to bias, variance, coverage, and MSE provides applied analysts with an interpretable decision aid for judging when mixed-effects 2SRI is likely to yield net gains over naïve survival analyses.

These conclusions are conditional on treatment-effect homogeneity. If treatment effects vary across studies, the performance of mixed-effects 2SRI, including its bias–variance profile, may differ. Evaluating performance under heterogeneous treatment effects remains an important direction for future methodological research.

## Supporting information

10.1017/rsm.2026.10103.sm001 Hufstedler et al. supplementary material Hufstedler et al. supplementary material

## Data Availability

Simulation code is available at https://github.com/hhufstedler/mixed-effects-2sri-simulation.

## Supplementary material

The supplementary material for this article can be found at https://doi.org/10.1017/rsm.2026.10103.

## Long descriptions

### Figure 2 Long description

The multi-panel visualization is organized into four main quadrants.

1. Top-left quadrant, Distribution of Estimates. A grid of histograms with beta-hat on the x-axis and count on the y-axis. 2 S R I distributions in pink remain centered on the dashed vertical truth line across all facets. Naive distributions in teal shift further to the right of the truth line as confounding increases from weak to very strong, particularly at higher I V strengths.

2. Top-right quadrant, Confidence Interval Coverage. A grid of plots showing fractional rank by absolute z on the y-axis and standardized C I on the x-axis. Blue areas represent coverage and red areas represent misses. 2 S R I panels show consistent blue coverage centered at zero. Naive panels show a rapid transition to solid red as confounding increases, indicating zero coverage as the intervals fail to capture the true effect.

3. Bottom-left quadrant, Estimates versus Standard Errors. Scatter plots with S E of beta-hat on the x-axis and beta-hat on the y-axis. 2 S R I points in pink show wider horizontal spread that narrows as I V strength increases from 0.3 to 1.0. Naive points in teal are tightly clustered but increasingly biased vertically away from the dashed truth line at negative 0.4 as confounding worsens.

4. Bottom-right quadrant, Method Comparison. Scatter plots comparing beta-hat naive on the x-axis to beta-hat 2 S R I on the y-axis. As confounding increases, the data clusters drift horizontally away from the diagonal dashed line of equality, while remaining vertically aligned with the horizontal dotted line representing the true theta of negative 0.4.

Navigate back to Figure 2.

### Figure 3 Long description

The multi-panel plot displays Relative bias percent on the x-axis, ranging from 0 to 150, and method on the y-axis.

Vertical Facets (Top Labels): Quintiles Q 1, Q 2, Q 3, Q 4, and Q 5 representing realized I V strength based on Wald chi-squared values.

Horizontal Facets (Right Labels): Confounding levels labeled weak, moderate, strong, and very strong.

Data Trends:

- Naive Method: Represented by a top dot in each panel. Bias increases significantly as confounding moves from weak to very strong. In the weak row, bias is near 25 percent. In the very strong row, bias exceeds 150 percent. The bias remains high and relatively constant across all Q quintiles within each confounding level.

- 2 S R I Method: Represented by a bottom dot in each panel. Bias is consistently lower than the Naive method. In the weak row, bias is near 0. In the very strong row, bias stabilizes around 50 percent. As I V strength increases from Q 1 to Q 5, the 95 percent Monte Carlo confidence interval bars for 2 S R I narrow significantly, indicating higher precision with stronger instruments.

Spatial Layout: Each panel contains two horizontal data points with vertical error bars. A dashed vertical line at 0 indicates the baseline for zero bias.

Navigate back to Figure 3.

### Figure 4 Long description

A multi-panel dot plot with 20 facets arranged in 4 rows and 5 columns.

Axes and Labels:

* The x-axis represents Coverage percentage from 0 percent to 100 percent with a vertical dashed line at the 95 percent target.

* The y-axis within each facet lists two methods: Naive at the top and 2 S R I below it.

* Top column facets are labeled Q 1, Q 2, Q 3, Q 4, and Q 5, representing Wald chi-squared quintiles.

* Right row facets are labeled by confounding strength: weak, moderate, strong, and very strong.

Data Trends:

* In the weak confounding row (top), both Naive and 2 S R I points cluster near the 95 percent dashed line across all quintiles Q 1 through Q 5.

* In the moderate, strong, and very strong confounding rows, the Naive method points drop sharply to near 0 percent coverage on the far left of every facet.

* In contrast, the 2 S R I method points remain consistently near the 95 percent target line across all confounding levels and all quintiles from Q 1 to Q 5.

* Each data point includes a small horizontal error bar representing the 95 percent Monte Carlo C I.

Navigate back to Figure 4.

### Figure 5 Long description

The multi-panel dot plot is organized into four rows representing unmeasured confounding levels (weak, moderate, strong, very strong) and five columns representing I V strength quintiles (Q 1, Q 2, Q 3, Q 4, Q 5). The x-axis for all panels is Empirical S E, with scales that vary by column. The y-axis in each panel lists two methods: Naive at the top and 2 S R I at the bottom.

* In the Q 1 column (weakest I V), Naive points are clustered near 0 on the x-axis, while 2 S R I points show the highest Empirical S E, positioned far to the right near 0.75 to 1.0.

* Moving from Q 1 to Q 5 (increasing I V strength), the 2 S R I points shift progressively leftward toward lower Empirical S E values. By Q 5, 2 S R I values are near 0.25.

* Across all rows (confounding levels), the Naive method consistently maintains a very low Empirical S E near the y-axis origin.

* Vertical error bars representing 95% M C C I s are visible on all points, appearing wider for 2 S R I in the Q 1 and Q 2 columns compared to the Naive method or stronger I V quintiles.

Navigate back to Figure 5.

### Figure 6 Long description

The grid is organized with four rows representing unmeasured confounding levels: weak, moderate, strong, and very strong. Five columns represent realized I V strength quintiles from Q 1 to Q 5. The x axis for all panels is M S E ranging from 0.00 to 0.40 or 0.75 depending on the column. The y axis lists the method as Naive or 2 S R I.

* In the weak confounding row, Naive M S E remains near zero across all quintiles. 2 S R I starts with high M S E and wide confidence intervals in Q 1 and Q 2, which decrease significantly as I V strength increases toward Q 5.

* In the moderate and strong confounding rows, Naive M S E increases to approximately 0.1 and 0.2 respectively. 2 S R I follows a similar trend of high initial M S E in Q 1 that drops below the Naive level by Q 4 or Q 5.

* In the very strong confounding row, Naive M S E is highest at approximately 0.4. 2 S R I starts with high M S E in Q 1 but rapidly declines, achieving a lower M S E than the Naive method starting from the Q 3 moderate instrument strength quintile.

Navigate back to Figure 6.

### Figure 7 Long description

The grid is organized into four columns representing design I V strengths of 0.3, 0.5, 0.8, and 1.0, and four rows representing confounding levels from weak at the top to very strong at the bottom. The y axis is labeled Wald chi super 2 d f equals 1 with a scale from 0 to 300. The x axis for each facet shows three categories: Pattern 1, Pattern 2, and Pattern 3.

* Moving from left to right across columns, the median Wald chi squared values increase significantly. At 0.3 strength, medians are near 25. At 0.5 strength, medians rise to approximately 60. At 0.8 strength, medians reach approximately 150. At 1.0 strength, medians are highest, exceeding 200.

* Moving from top to bottom across rows, there is a slight downward shift in the distribution as confounding increases from weak to very strong, though the overall shape of the violin plots remains consistent.

* Within each individual facet, the three allocation patterns show nearly identical distributions, with Pattern 1, Pattern 2, and Pattern 3 exhibiting similar medians and interquartile ranges.

* The vertical spread of the violin plots increases as the I V strength increases, indicating greater variance at higher correlation levels.

Navigate back to Figure 7.
