# Supplementary material for: Mixed-effects two-stage residual inclusion methods for individual patient data meta-analysis: A methodological framework for causal inference in survival analysis
Source: Res Synth Methods. 2026 Jun 29;17(6):1158–75. doi: 10.1017/rsm.2026.10103 (PMC13732372; doi:10.1017/rsm.2026.10103)
Supplement: Supplementary file 1 [file S1759287926101033sup001.pdf]

Appendix A: Additional Figures and Tables

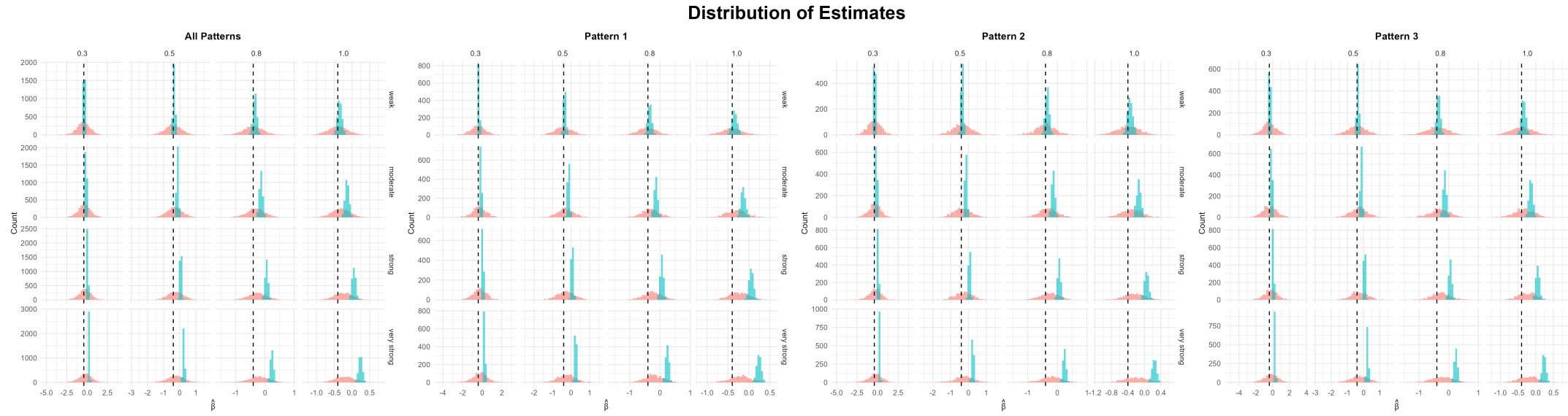

Figure A1: Distribution of parameter estimates by IV allocation pattern. Left: All patterns combined. Remaining panels: Individual patterns 1, 2, and 3. Results stratified by IV strength (columns) and confounding level (rows).

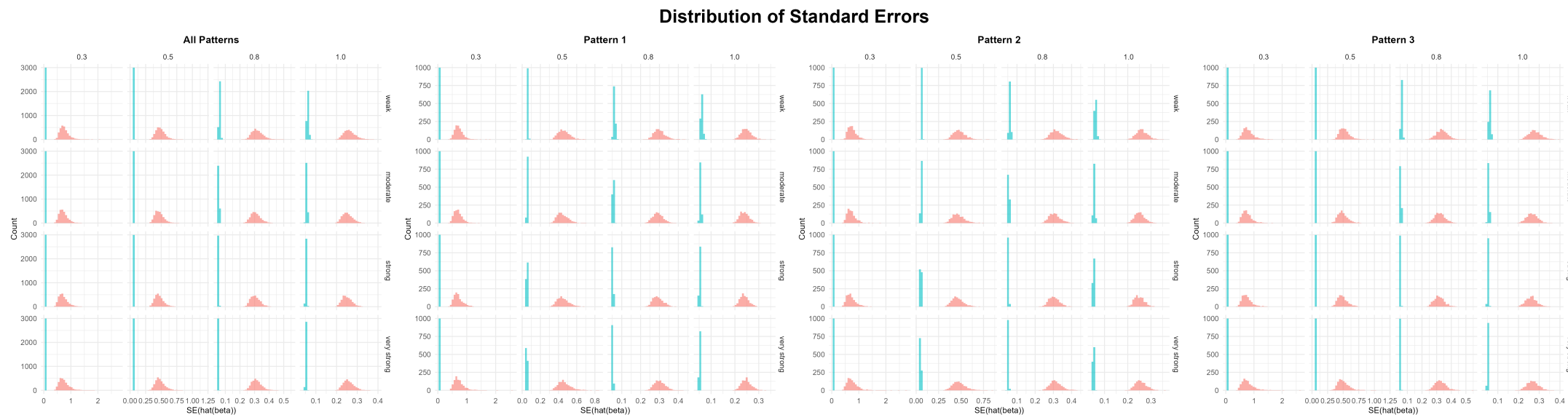

Figure A2: Distribution of standard errors by IV allocation pattern. Left: All patterns combined. Remaining panels: Individual patterns 1, 2, and 3. Results stratified by IV strength (columns) and confounding level (rows).

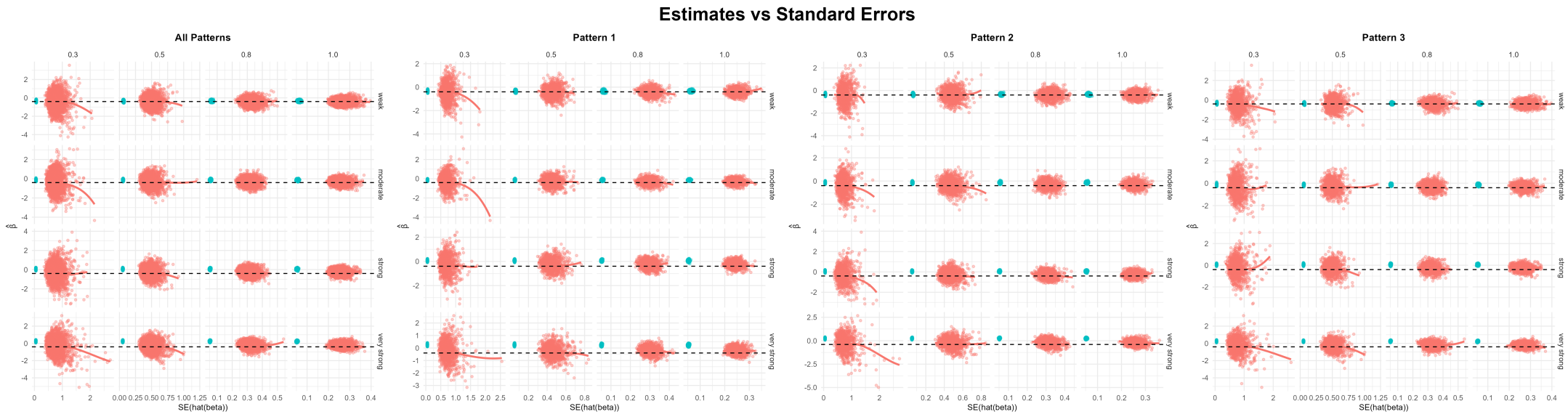

Figure A3: Relationship between parameter estimates and standard errors by IV allocation pattern. Left: All patterns combined. Remaining panels: Individual patterns 1, 2, and 3. Demonstrates weak-instrument pathology (trumpet shape) for 2SRI versus stable precision for naïve estimation.

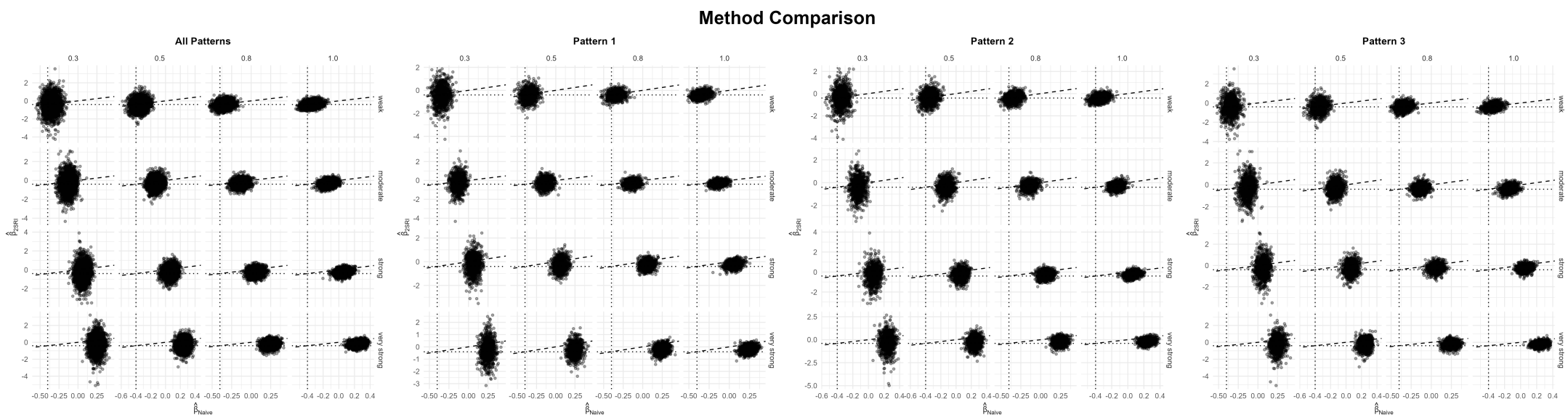

Figure A4: Method-to-method comparison of parameter estimates by IV allocation pattern. Left: All patterns combined. Remaining panels: Individual patterns 1, 2, and 3. Diagonal reference line indicates perfect agreement between methods.

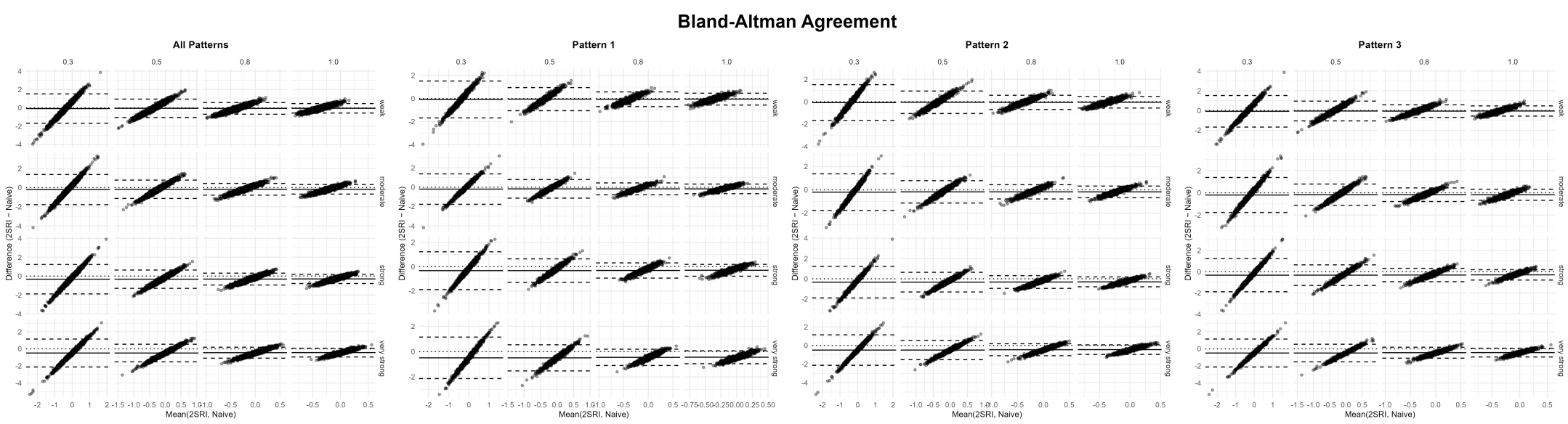

Figure A5: Bland-Altman agreement analysis by IV allocation pattern. Left: All patterns combined. Remaining panels: Individual patterns 1, 2, and 3. Shows difference between methods versus their mean, with limits of agreement.

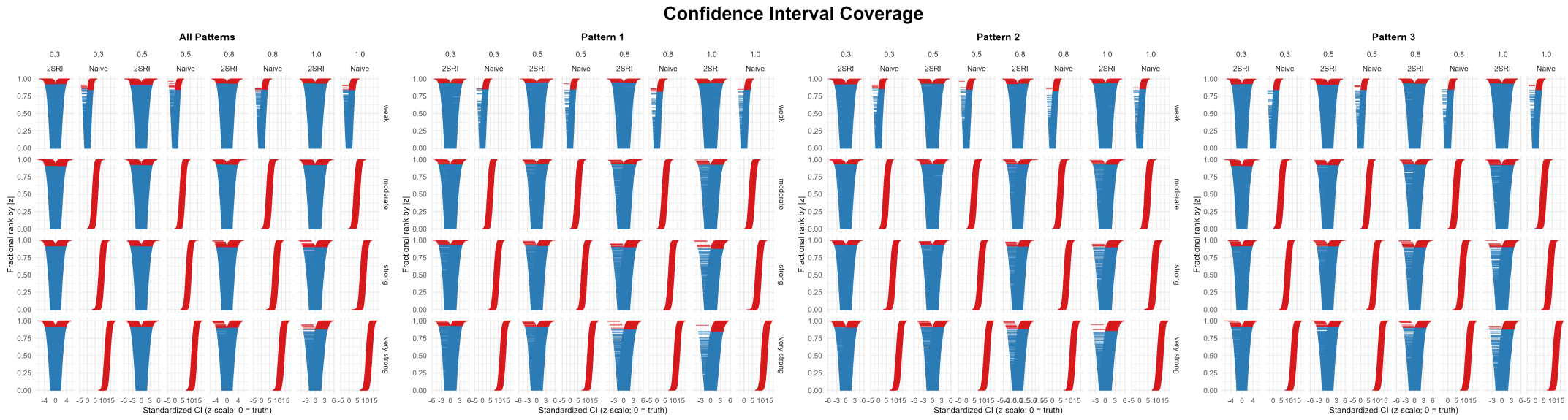

Figure A6: Confidence interval coverage assessment by IV allocation pattern. Left: All patterns combined. Remaining panels: Individual patterns 1, 2, and 3. Confidence intervals ranked by |z-score| with coverage indicated by color (blue = covers truth, red = misses truth).

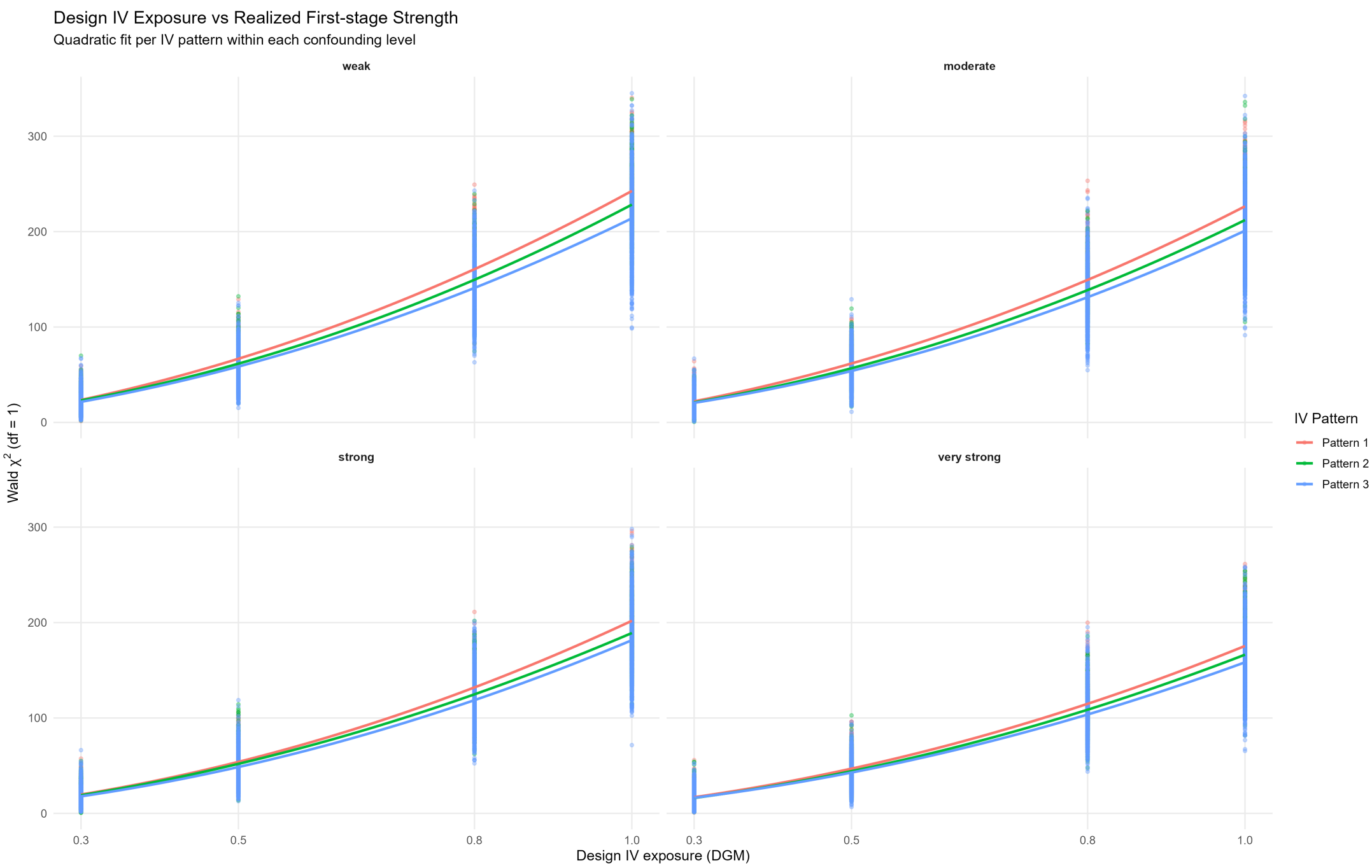

Figure A7: Design parameters to realized IV strength. Instrument–exposure correlation (x-axis) versus realized IV strength (Wald χ^2; df=1) (y-axis), with panels by unmeasured confounding and lines for IV allocation patterns. Realized IV strength increases smoothly with the design's IV–exposure correlation, with only modest shifts across allocation patterns.

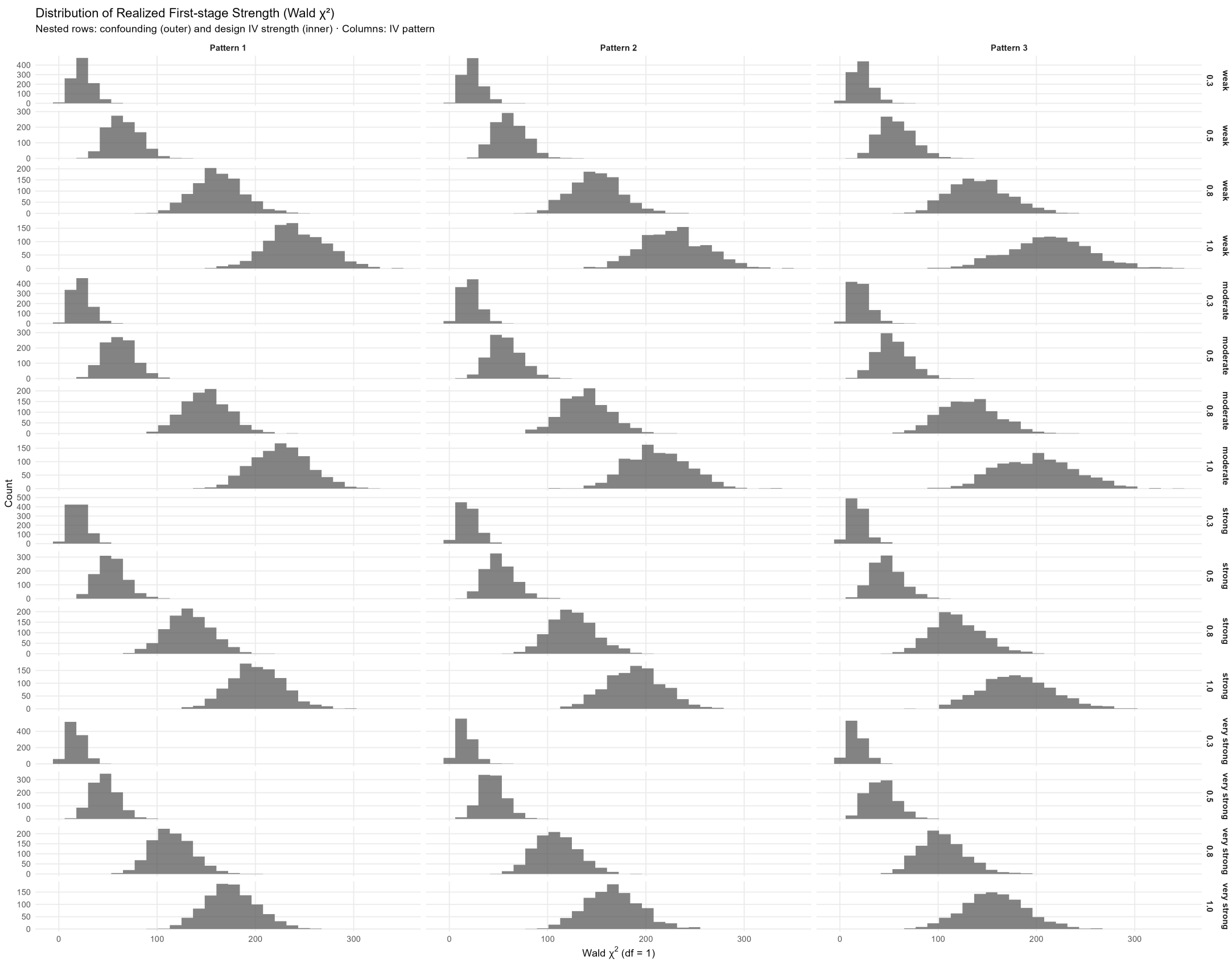

Figure A8: Histograms of realized IV strength (Wald χ^2; df=1) across all simulation scenarios. Top facets show IV allocation patterns (1–3); nested row facets show unmeasured confounding (outer strip) with IV–exposure strength (inner strip). There is a clear progression from weak to strong instruments as IV–exposure correlation increases, largely consistent across allocation patterns.

Table A1: Performance measures by confounding level and IV strength (design). Entries are point estimates with Monte Carlo standard errors in parentheses.

Scenario			Performance measures (value; MCSE)					Reps
Confounding	IV strength (design)	Method	Rel. bias (%)	Empirical SE	MSE	Precision (CI width)	Coverage	n
weak	0.3	2SRI	6.4 (3.7)	0.817 (0.011)	0.669 (0.021)	2.970 (0.012)	93.1% (0.5%)	3000
weak	0.3	Naive	13.8 (0.3)	0.062 (0.001)	0.007 (0.000)	0.240 (0.000)	85.3% (0.6%)	3000
weak	0.5	2SRI	0.4 (2.4)	0.517 (0.007)	0.267 (0.008)	1.907 (0.005)	93.5% (0.4%)	3000
weak	0.5	Naive	13.6 (0.3)	0.063 (0.001)	0.007 (0.000)	0.240 (0.000)	85.0% (0.7%)	3000
weak	0.8	2SRI	0.9 (1.5)	0.331 (0.004)	0.110 (0.003)	1.250 (0.003)	93.9% (0.4%)	3000
weak	0.8	Naive	14.6 (0.3)	0.062 (0.001)	0.007 (0.000)	0.239 (0.000)	83.3% (0.7%)	3000
weak	1.0	2SRI	1.0 (1.2)	0.272 (0.004)	0.074 (0.002)	1.030 (0.002)	94.0% (0.4%)	3000
weak	1.0	Naive	13.7 (0.3)	0.062 (0.001)	0.007 (0.000)	0.239 (0.000)	85.0% (0.7%)	3000
moderate	0.3	2SRI	16.9 (3.7)	0.804 (0.010)	0.651 (0.020)	2.853 (0.012)	92.9% (0.5%)	3000
moderate	0.3	Naive	67.0 (0.2)	0.055 (0.001)	0.075 (0.001)	0.214 (0.000)	0.2% (0.1%)	3000
moderate	0.5	2SRI	22.5 (2.2)	0.492 (0.006)	0.250 (0.007)	1.841 (0.005)	93.4% (0.5%)	3000
moderate	0.5	Naive	66.3 (0.3)	0.055 (0.001)	0.073 (0.001)	0.215 (0.000)	0.3% (0.1%)	3000
moderate	0.8	2SRI	22.7 (1.4)	0.316 (0.004)	0.108 (0.003)	1.204 (0.002)	93.5% (0.4%)	3000
moderate	0.8	Naive	66.2 (0.2)	0.055 (0.001)	0.073 (0.001)	0.215 (0.000)	0.3% (0.1%)	3000
moderate	1.0	2SRI	21.4 (1.2)	0.260 (0.003)	0.075 (0.002)	0.990 (0.002)	93.1% (0.5%)	3000
moderate	1.0	Naive	65.6 (0.2)	0.054 (0.001)	0.072 (0.001)	0.217 (0.000)	0.3% (0.1%)	3000
strong	0.3	2SRI	33.9 (3.6)	0.794 (0.010)	0.648 (0.021)	2.854 (0.014)	93.0% (0.5%)	3000
strong	0.3	Naive	116.3 (0.2)	0.052 (0.001)	0.219 (0.001)	0.201 (0.000)	0.0% (0.0%)	3000
strong	0.5	2SRI	33.6 (2.3)	0.496 (0.006)	0.264 (0.007)	1.833 (0.005)	92.6% (0.5%)	3000
strong	0.5	Naive	115.6 (0.2)	0.051 (0.001)	0.216 (0.001)	0.203 (0.000)	0.0% (0.0%)	3000
strong	0.8	2SRI	35.5 (1.5)	0.321 (0.004)	0.123 (0.003)	1.195 (0.002)	90.8% (0.5%)	3000
strong	0.8	Naive	114.7 (0.2)	0.052 (0.001)	0.213 (0.001)	0.205 (0.000)	0.0% (0.0%)	3000
strong	1.0	2SRI	38.6 (1.2)	0.254 (0.003)	0.088 (0.002)	0.981 (0.002)	89.7% (0.6%)	3000
strong	1.0	Naive	113.6 (0.2)	0.053 (0.001)	0.209 (0.001)	0.207 (0.000)	0.0% (0.0%)	3000
very strong	0.3	2SRI	38.6 (3.8)	0.837 (0.011)	0.724 (0.024)	2.974 (0.016)	92.0% (0.5%)	3000
very strong	0.3	Naive	161.8 (0.2)	0.051 (0.001)	0.421 (0.001)	0.197 (0.000)	0.0% (0.0%)	3000
very strong	0.5	2SRI	38.2 (2.4)	0.528 (0.007)	0.302 (0.008)	1.893 (0.006)	91.6% (0.5%)	3000
very strong	0.5	Naive	161.5 (0.2)	0.050 (0.001)	0.420 (0.001)	0.199 (0.000)	0.0% (0.0%)	3000

Table A1: Performance measures by confounding level and IV strength (design). Entries are point estimates with Monte Carlo standard errors in parentheses. *(continued)*

Scenario			Performance measures (value; MCSE)					Reps
Confounding	IV strength (design)	Method	Rel. bias (%)	Empirical SE	MSE	Precision (CI width)	Coverage	n
very strong	0.8	2SRI	46.8 (1.5)	0.330 (0.004)	0.144 (0.004)	1.230 (0.002)	89.3% (0.6%)	3000
very strong	0.8	Naive	160.4 (0.2)	0.052 (0.001)	0.414 (0.001)	0.203 (0.000)	0.0% (0.0%)	3000
very strong	1.0	2SRI	49.1 (1.2)	0.266 (0.003)	0.109 (0.003)	1.010 (0.002)	86.5% (0.6%)	3000
very strong	1.0	Naive	159.8 (0.2)	0.053 (0.001)	0.412 (0.001)	0.206 (0.000)	0.0% (0.0%)	3000

Table A2: Performance measures (weak confounding). Entries are point estimates with Monte Carlo standard errors in parentheses.

Scenario			Performance measures (value; MCSE)					Reps
IV strength (design)	IV pattern	Method	Rel. bias (%)	Empirical SE	MSE	Precision (CI width)	Coverage	n
0.3	1	2SRI	10.7 (6.1)	0.770 (0.017)	0.594 (0.031)	2.845 (0.019)	94.0% (0.8%)	1000
0.3	1	Naive	14.0 (0.5)	0.061 (0.001)	0.007 (0.000)	0.240 (0.000)	85.5% (1.1%)	1000
0.3	2	2SRI	3.1 (6.5)	0.827 (0.019)	0.683 (0.036)	2.946 (0.020)	92.5% (0.8%)	1000
0.3	2	Naive	13.4 (0.5)	0.062 (0.001)	0.007 (0.000)	0.241 (0.000)	85.9% (1.1%)	1000
0.3	3	2SRI	5.5 (6.7)	0.854 (0.019)	0.729 (0.040)	3.118 (0.024)	92.8% (0.8%)	1000
0.3	3	Naive	14.1 (0.5)	0.062 (0.001)	0.007 (0.000)	0.240 (0.000)	84.6% (1.1%)	1000
0.5	1	2SRI	4.7 (3.7)	0.472 (0.011)	0.223 (0.011)	1.799 (0.008)	95.0% (0.7%)	1000
0.5	1	Naive	13.6 (0.5)	0.061 (0.001)	0.007 (0.000)	0.239 (0.000)	85.2% (1.1%)	1000
0.5	2	2SRI	7.0 (4.1)	0.514 (0.012)	0.265 (0.013)	1.887 (0.008)	93.5% (0.8%)	1000
0.5	2	Naive	13.6 (0.5)	0.063 (0.001)	0.007 (0.000)	0.240 (0.000)	85.1% (1.1%)	1000
0.5	3	2SRI	1.3 (4.4)	0.561 (0.013)	0.314 (0.016)	2.035 (0.010)	92.1% (0.9%)	1000
0.5	3	Naive	13.6 (0.5)	0.063 (0.001)	0.007 (0.000)	0.240 (0.000)	84.7% (1.1%)	1000
0.8	1	2SRI	4.8 (2.4)	0.298 (0.007)	0.089 (0.004)	1.177 (0.004)	95.1% (0.7%)	1000
0.8	1	Naive	14.8 (0.5)	0.063 (0.001)	0.007 (0.000)	0.239 (0.001)	82.0% (1.2%)	1000
0.8	2	2SRI	0.4 (2.6)	0.331 (0.007)	0.109 (0.005)	1.248 (0.004)	93.1% (0.8%)	1000
0.8	2	Naive	14.8 (0.5)	0.063 (0.001)	0.007 (0.000)	0.239 (0.000)	82.9% (1.2%)	1000
0.8	3	2SRI	1.6 (2.9)	0.362 (0.008)	0.131 (0.006)	1.325 (0.005)	93.6% (0.8%)	1000
0.8	3	Naive	14.3 (0.5)	0.060 (0.001)	0.007 (0.000)	0.240 (0.001)	84.9% (1.1%)	1000
1.0	1	2SRI	0.6 (2.0)	0.248 (0.006)	0.062 (0.003)	0.971 (0.003)	94.1% (0.7%)	1000
1.0	1	Naive	13.7 (0.5)	0.062 (0.001)	0.007 (0.000)	0.238 (0.000)	84.8% (1.1%)	1000
1.0	2	2SRI	0.7 (2.1)	0.269 (0.006)	0.073 (0.003)	1.023 (0.003)	94.1% (0.7%)	1000
1.0	2	Naive	13.5 (0.5)	0.061 (0.001)	0.007 (0.000)	0.240 (0.000)	85.9% (1.1%)	1000
1.0	3	2SRI	3.0 (2.3)	0.295 (0.007)	0.087 (0.004)	1.097 (0.004)	93.7% (0.8%)	1000
1.0	3	Naive	13.8 (0.5)	0.062 (0.001)	0.007 (0.000)	0.239 (0.000)	84.4% (1.1%)	1000

Table A3: Performance measures (moderate confounding). Entries are point estimates with Monte Carlo standard errors in parentheses.

Scenario			Performance measures (value; MCSE)					Reps
IV strength (design)	IV pattern	Method	Rel. bias (%)	Empirical SE	MSE	Precision (CI width)	Coverage	n
0.3	1	2SRI	26.9 (5.9)	0.744 (0.017)	0.565 (0.032)	2.747 (0.020)	93.6% (0.8%)	1000
0.3	1	Naive	67.0 (0.4)	0.053 (0.001)	0.075 (0.001)	0.214 (0.000)	0.1% (0.1%)	1000
0.3	2	2SRI	9.9 (6.1)	0.774 (0.017)	0.599 (0.031)	2.843 (0.021)	94.0% (0.8%)	1000
0.3	2	Naive	66.9 (0.4)	0.054 (0.001)	0.074 (0.001)	0.214 (0.000)	0.2% (0.1%)	1000
0.3	3	2SRI	14.0 (7.0)	0.887 (0.020)	0.789 (0.040)	2.968 (0.023)	91.2% (0.9%)	1000
0.3	3	Naive	67.1 (0.4)	0.057 (0.001)	0.075 (0.001)	0.214 (0.000)	0.4% (0.2%)	1000
0.5	1	2SRI	24.8 (3.5)	0.438 (0.010)	0.202 (0.009)	1.744 (0.008)	94.1% (0.7%)	1000
0.5	1	Naive	66.4 (0.4)	0.054 (0.001)	0.073 (0.001)	0.214 (0.000)	0.3% (0.2%)	1000
0.5	2	2SRI	13.1 (4.0)	0.503 (0.011)	0.255 (0.012)	1.840 (0.009)	93.6% (0.8%)	1000
0.5	2	Naive	66.4 (0.4)	0.056 (0.001)	0.074 (0.001)	0.215 (0.000)	0.2% (0.1%)	1000
0.5	3	2SRI	29.6 (4.2)	0.529 (0.012)	0.294 (0.014)	1.938 (0.010)	92.5% (0.8%)	1000
0.5	3	Naive	66.1 (0.4)	0.056 (0.001)	0.073 (0.001)	0.215 (0.000)	0.3% (0.2%)	1000
0.8	1	2SRI	20.3 (2.3)	0.293 (0.007)	0.092 (0.004)	1.145 (0.003)	93.9% (0.8%)	1000
0.8	1	Naive	66.4 (0.4)	0.056 (0.001)	0.074 (0.001)	0.216 (0.000)	0.2% (0.1%)	1000
0.8	2	2SRI	21.8 (2.5)	0.315 (0.007)	0.107 (0.005)	1.204 (0.004)	93.5% (0.8%)	1000
0.8	2	Naive	65.5 (0.4)	0.055 (0.001)	0.072 (0.001)	0.216 (0.000)	0.5% (0.2%)	1000
0.8	3	2SRI	26.1 (2.7)	0.337 (0.008)	0.124 (0.006)	1.263 (0.004)	93.2% (0.8%)	1000
0.8	3	Naive	66.7 (0.4)	0.053 (0.001)	0.074 (0.001)	0.215 (0.000)	0.2% (0.1%)	1000
1.0	1	2SRI	23.7 (1.9)	0.237 (0.005)	0.065 (0.003)	0.941 (0.002)	93.1% (0.8%)	1000
1.0	1	Naive	65.6 (0.4)	0.054 (0.001)	0.072 (0.001)	0.216 (0.000)	0.2% (0.1%)	1000
1.0	2	2SRI	22.9 (2.0)	0.259 (0.006)	0.076 (0.004)	0.985 (0.003)	93.5% (0.8%)	1000
1.0	2	Naive	65.9 (0.4)	0.053 (0.001)	0.072 (0.001)	0.217 (0.000)	0.1% (0.1%)	1000
1.0	3	2SRI	17.6 (2.2)	0.281 (0.006)	0.084 (0.004)	1.044 (0.003)	92.6% (0.8%)	1000
1.0	3	Naive	65.4 (0.4)	0.055 (0.001)	0.071 (0.001)	0.217 (0.000)	0.6% (0.2%)	1000

Table A4: Performance measures (strong confounding). Entries are point estimates with Monte Carlo standard errors in parentheses.

Scenario			Performance measures (value; MCSE)					Reps
IV strength (design)	IV pattern	Method	Rel. bias (%)	Empirical SE	MSE	Precision (CI width)	Coverage	n
0.3	1	2SRI	34.9 (5.9)	0.742 (0.017)	0.569 (0.030)	2.765 (0.020)	93.8% (0.8%)	1000
0.3	1	Naive	116.1 (0.4)	0.053 (0.001)	0.219 (0.002)	0.201 (0.000)	0.0% (0.0%)	1000
0.3	2	2SRI	25.0 (6.3)	0.793 (0.018)	0.637 (0.036)	2.832 (0.024)	93.3% (0.8%)	1000
0.3	2	Naive	116.8 (0.4)	0.053 (0.001)	0.221 (0.002)	0.201 (0.000)	0.0% (0.0%)	1000
0.3	3	2SRI	41.6 (6.7)	0.843 (0.019)	0.738 (0.040)	2.966 (0.025)	91.9% (0.9%)	1000
0.3	3	Naive	115.9 (0.4)	0.052 (0.001)	0.218 (0.002)	0.201 (0.000)	0.0% (0.0%)	1000
0.5	1	2SRI	31.9 (3.7)	0.473 (0.011)	0.240 (0.011)	1.760 (0.008)	92.4% (0.8%)	1000
0.5	1	Naive	115.5 (0.4)	0.051 (0.001)	0.216 (0.001)	0.203 (0.000)	0.0% (0.0%)	1000
0.5	2	2SRI	32.4 (3.8)	0.483 (0.011)	0.250 (0.011)	1.814 (0.008)	93.7% (0.8%)	1000
0.5	2	Naive	115.4 (0.4)	0.053 (0.001)	0.216 (0.002)	0.203 (0.000)	0.0% (0.0%)	1000
0.5	3	2SRI	36.4 (4.2)	0.530 (0.012)	0.302 (0.014)	1.924 (0.010)	91.8% (0.9%)	1000
0.5	3	Naive	115.9 (0.4)	0.051 (0.001)	0.218 (0.001)	0.202 (0.000)	0.0% (0.0%)	1000
0.8	1	2SRI	37.5 (2.4)	0.307 (0.007)	0.117 (0.005)	1.148 (0.003)	90.4% (0.9%)	1000
0.8	1	Naive	115.2 (0.4)	0.051 (0.001)	0.215 (0.002)	0.205 (0.000)	0.0% (0.0%)	1000
0.8	2	2SRI	33.2 (2.5)	0.319 (0.007)	0.120 (0.005)	1.192 (0.004)	91.4% (0.9%)	1000
0.8	2	Naive	114.9 (0.4)	0.051 (0.001)	0.214 (0.001)	0.205 (0.000)	0.0% (0.0%)	1000
0.8	3	2SRI	35.8 (2.6)	0.335 (0.007)	0.132 (0.006)	1.244 (0.004)	90.5% (0.9%)	1000
0.8	3	Naive	113.9 (0.4)	0.053 (0.001)	0.210 (0.002)	0.204 (0.000)	0.0% (0.0%)	1000
1.0	1	2SRI	38.8 (2.0)	0.247 (0.006)	0.085 (0.004)	0.939 (0.002)	88.2% (1.0%)	1000
1.0	1	Naive	113.6 (0.4)	0.055 (0.001)	0.210 (0.002)	0.207 (0.000)	0.0% (0.0%)	1000
1.0	2	2SRI	38.8 (2.0)	0.252 (0.006)	0.088 (0.004)	0.979 (0.003)	90.4% (0.9%)	1000
1.0	2	Naive	113.9 (0.4)	0.054 (0.001)	0.210 (0.002)	0.208 (0.000)	0.0% (0.0%)	1000
1.0	3	2SRI	38.1 (2.1)	0.264 (0.006)	0.093 (0.004)	1.026 (0.003)	90.6% (0.9%)	1000
1.0	3	Naive	113.4 (0.4)	0.050 (0.001)	0.208 (0.001)	0.207 (0.000)	0.0% (0.0%)	1000

Table A5: Performance measures (very strong confounding). Entries are point estimates with Monte Carlo standard errors in parentheses.

Scenario			Performance measures (value; MCSE)					Reps
IV strength (design)	IV pattern	Method	Rel. bias (%)	Empirical SE	MSE	Precision (CI width)	Coverage	n
0.3	1	2SRI	44.9 (6.1)	0.772 (0.017)	0.628 (0.032)	2.882 (0.024)	93.4% (0.8%)	1000
0.3	1	Naive	161.8 (0.4)	0.049 (0.001)	0.422 (0.002)	0.197 (0.000)	0.0% (0.0%)	1000
0.3	2	2SRI	28.5 (6.9)	0.867 (0.019)	0.763 (0.046)	2.997 (0.028)	91.0% (0.9%)	1000
0.3	2	Naive	161.5 (0.4)	0.051 (0.001)	0.420 (0.002)	0.197 (0.000)	0.0% (0.0%)	1000
0.3	3	2SRI	42.4 (6.9)	0.867 (0.019)	0.779 (0.046)	3.043 (0.028)	91.6% (0.9%)	1000
0.3	3	Naive	162.0 (0.4)	0.052 (0.001)	0.423 (0.002)	0.196 (0.000)	0.0% (0.0%)	1000
0.5	1	2SRI	38.2 (3.9)	0.498 (0.011)	0.271 (0.013)	1.823 (0.009)	91.6% (0.9%)	1000
0.5	1	Naive	161.9 (0.4)	0.050 (0.001)	0.422 (0.002)	0.199 (0.000)	0.0% (0.0%)	1000
0.5	2	2SRI	37.7 (4.1)	0.525 (0.012)	0.298 (0.014)	1.881 (0.009)	91.4% (0.9%)	1000
0.5	2	Naive	161.2 (0.4)	0.050 (0.001)	0.418 (0.002)	0.199 (0.000)	0.0% (0.0%)	1000
0.5	3	2SRI	38.9 (4.4)	0.561 (0.013)	0.338 (0.016)	1.976 (0.011)	91.8% (0.9%)	1000
0.5	3	Naive	161.4 (0.4)	0.049 (0.001)	0.419 (0.002)	0.199 (0.000)	0.0% (0.0%)	1000
0.8	1	2SRI	49.3 (2.4)	0.304 (0.007)	0.131 (0.006)	1.184 (0.004)	88.4% (1.0%)	1000
0.8	1	Naive	159.8 (0.4)	0.052 (0.001)	0.411 (0.002)	0.203 (0.000)	0.0% (0.0%)	1000
0.8	2	2SRI	47.6 (2.7)	0.338 (0.008)	0.150 (0.006)	1.226 (0.004)	88.8% (1.0%)	1000
0.8	2	Naive	160.3 (0.4)	0.050 (0.001)	0.414 (0.002)	0.203 (0.000)	0.0% (0.0%)	1000
0.8	3	2SRI	43.6 (2.7)	0.348 (0.008)	0.151 (0.006)	1.280 (0.005)	90.7% (0.9%)	1000
0.8	3	Naive	161.0 (0.4)	0.053 (0.001)	0.417 (0.002)	0.202 (0.000)	0.0% (0.0%)	1000
1.0	1	2SRI	50.5 (2.0)	0.255 (0.006)	0.106 (0.004)	0.971 (0.003)	85.1% (1.1%)	1000
1.0	1	Naive	159.7 (0.4)	0.054 (0.001)	0.411 (0.002)	0.206 (0.000)	0.0% (0.0%)	1000
1.0	2	2SRI	47.6 (2.1)	0.270 (0.006)	0.109 (0.005)	1.004 (0.003)	86.2% (1.1%)	1000
1.0	2	Naive	160.4 (0.4)	0.052 (0.001)	0.414 (0.002)	0.206 (0.000)	0.0% (0.0%)	1000
1.0	3	2SRI	49.2 (2.2)	0.273 (0.006)	0.113 (0.005)	1.053 (0.003)	88.2% (1.0%)	1000
1.0	3	Naive	159.4 (0.4)	0.052 (0.001)	0.409 (0.002)	0.205 (0.000)	0.0% (0.0%)	1000

Appendix B: Sensitivity to Exclusion and Independence Violations

The primary simulations examined the performance of 2SRI across varying levels of instrument relevance. Here, we assess sensitivity to violations of the remaining two assumptions: the exclusion restriction and the independence assumption. These simulations were implemented as targeted stress tests and therefore fixed the instrument–treatment association at 0.8 on the log-odds scale with balanced instrument allocation (50%), under strong and very strong unmeasured confounding, where 2SRI performed well in the main results. All other elements of the data-generating process and analysis models were identical to those used in the primary simulations.

Exclusion restriction violation.

To violate the exclusion restriction, we introduced a direct effect of the instrument on the event process that was not mediated by treatment. Specifically, a nonzero coefficient ($\gamma = 0.08$) for the instrument was added to the cause-specific log-hazard of death and, symmetrically, with opposite sign to the cause-specific log-hazard of discharge:

$$\log h_{ijk}^{\text{death}}(t) = \cdots + \beta_T T_{ijk} + \beta_U^{(S)} U_i + \gamma Z_{ijk}, \tag{0.1}$$

$$\log h_{ijk}^{\text{discharge}}(t) = \cdots - \beta_T T_{ijk} - \beta_U^{(S)} U_i - \gamma Z_{ijk}. \tag{0.2}$$

This construction induces a direct pathway from the instrument to the observed time-to-event outcome, thereby violating the exclusion restriction.

Independence violation.

To violate the independence assumption, the instrument was generated to depend directly on the unmeasured confounder. Let p_j denote the study-specific baseline probability of instrument assignment. The instrument was generated according to

$$Z_{ijk} \sim \text{Bernoulli}\Big(\text{logit}^{-1}\big(\text{logit}(p_j) + \alpha U_i\big)\Big), \tag{0.3}$$

with $\alpha = 0.2$. This preserves the marginal study-level instrument prevalence while inducing correlation between the instrument and the unmeasured confounder, violating the independence assumption.

Figures B1–B2 report bias, empirical standard error, coverage, and mean squared error under strong and very strong confounding, respectively. Table B1 summarizes these results.

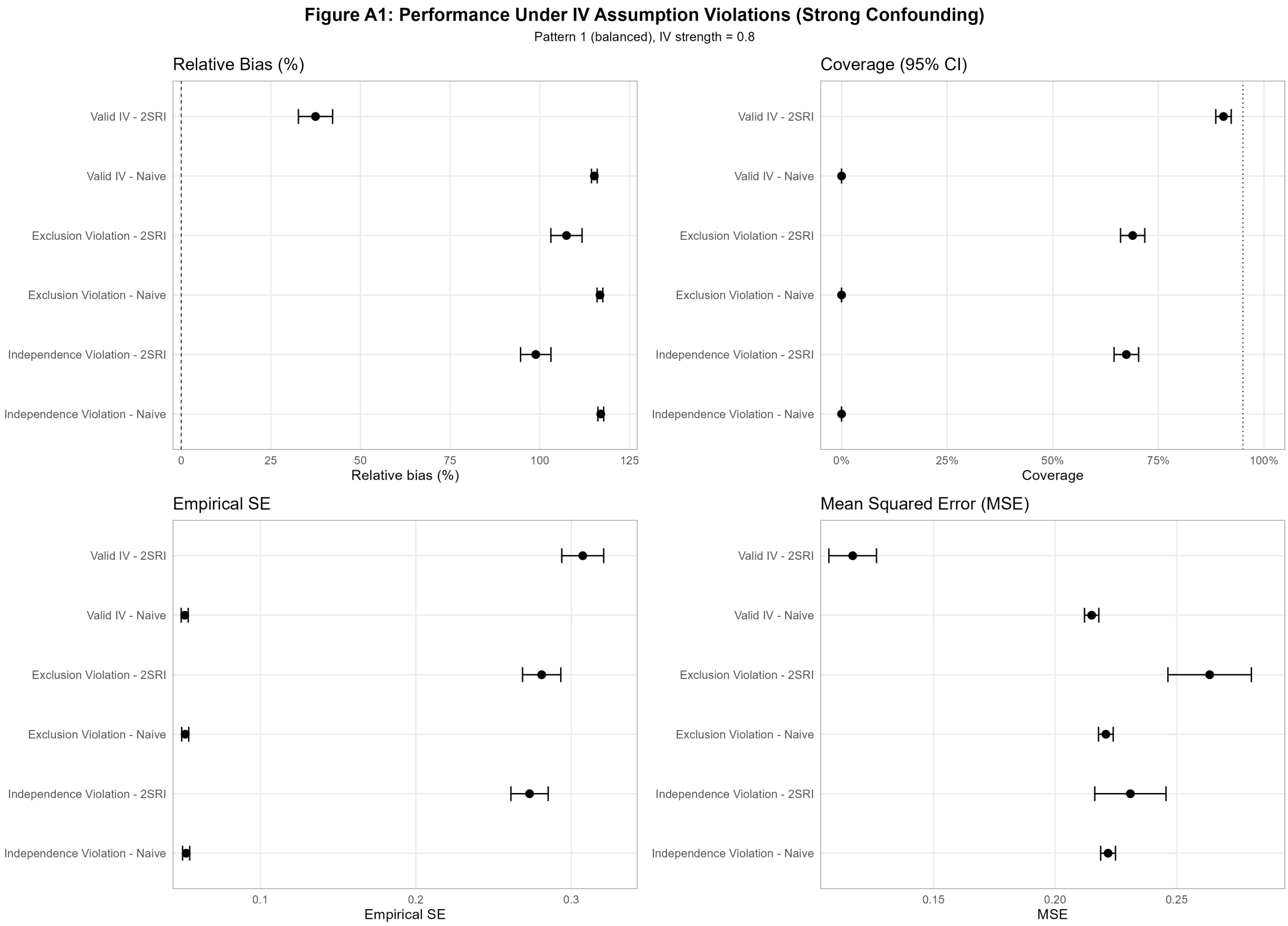

Figure B1: Performance under IV assumption violations (strong confounding). Relative bias, coverage, empirical SE, and MSE for 2SRI and naïve estimators under valid IV, exclusion restriction violation, and independence violation. IV strength = 0.8, balanced allocation.

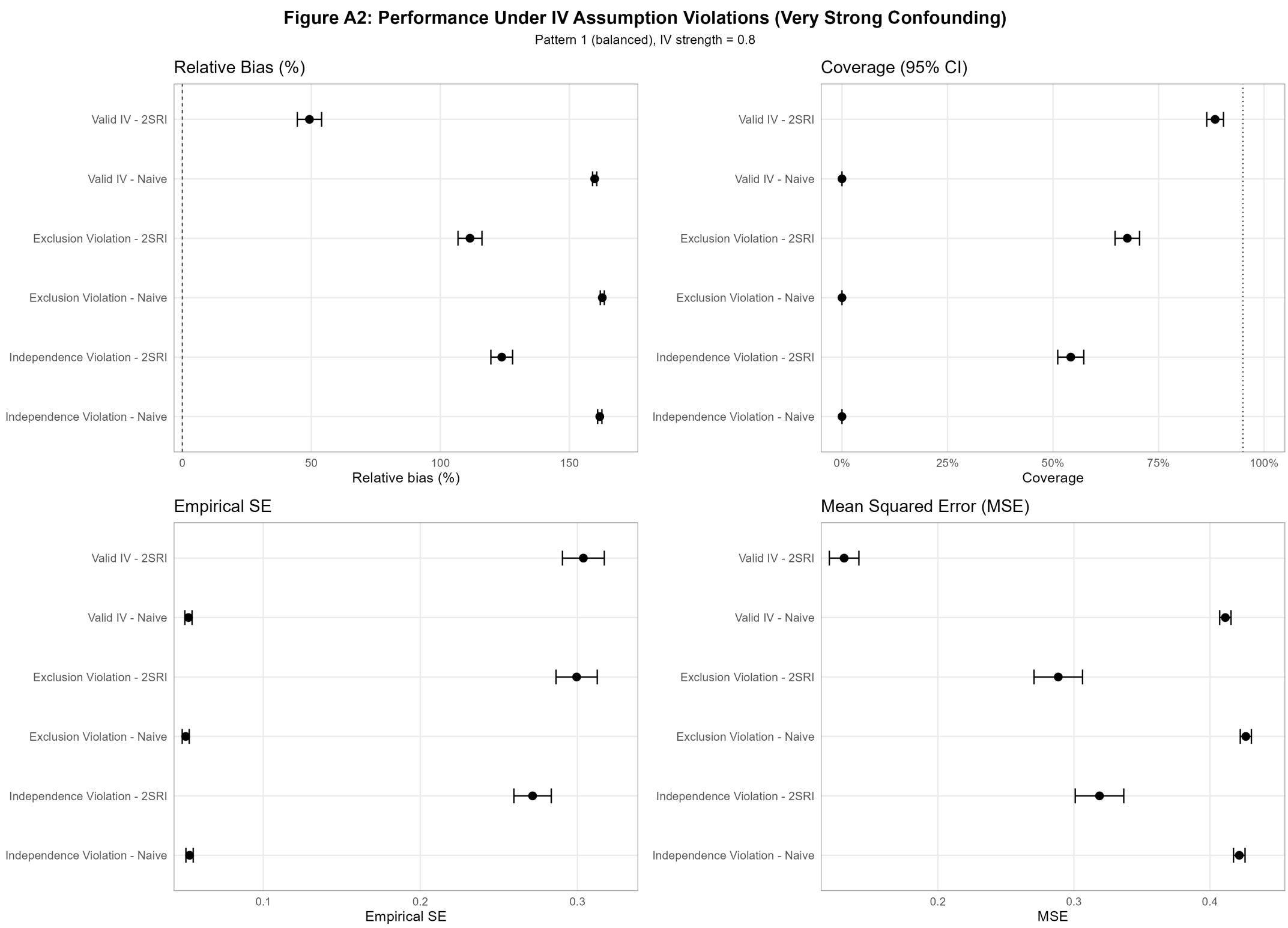

Figure B2: Performance under IV assumption violations (very strong confounding). Relative bias, coverage, empirical SE, and MSE for 2SRI and naïve estimators under valid IV, exclusion restriction violation, and independence violation. IV strength = 0.8, balanced allocation.

Table B1: Performance under IV assumption violations (Pattern 1, IV strength = 0.8). Values show point estimates with Monte Carlo SEs in parentheses.

Scenario			Performance (value; MCSE)				
Confounding	Assumption	Method	Rel. Bias (%)	Emp. SE	MSE	Coverage	n
strong	Valid IV	2SRI	37.5 (2.4)	0.307 (0.007)	0.117 (0.005)	90.4% (0.9%)	1000
strong	Valid IV	Naive	115.2 (0.4)	0.051 (0.001)	0.215 (0.002)	0.0% (0.0%)	1000
strong	Exclusion Violation	2SRI	107.4 (2.2)	0.281 (0.006)	0.264 (0.009)	68.9% (1.5%)	1000
strong	Exclusion Violation	Naive	116.8 (0.4)	0.052 (0.001)	0.221 (0.002)	0.0% (0.0%)	1000
strong	Independence Violation	2SRI	98.9 (2.2)	0.273 (0.006)	0.231 (0.007)	67.4% (1.5%)	1000
strong	Independence Violation	Naive	117.0 (0.4)	0.052 (0.001)	0.222 (0.002)	0.0% (0.0%)	1000
very strong	Valid IV	2SRI	49.3 (2.4)	0.304 (0.007)	0.131 (0.006)	88.4% (1.0%)	1000
very strong	Valid IV	Naive	159.8 (0.4)	0.052 (0.001)	0.411 (0.002)	0.0% (0.0%)	1000
very strong	Exclusion Violation	2SRI	111.5 (2.4)	0.299 (0.007)	0.289 (0.009)	67.6% (1.5%)	1000
very strong	Exclusion Violation	Naive	162.8 (0.4)	0.051 (0.001)	0.426 (0.002)	0.0% (0.0%)	1000
very strong	Independence Violation	2SRI	123.8 (2.1)	0.271 (0.006)	0.319 (0.009)	54.2% (1.6%)	1000
very strong	Independence Violation	Naive	161.8 (0.4)	0.053 (0.001)	0.422 (0.002)	0.0% (0.0%)	1000
